# Stochastic Dynamics Underlying Cognitive Stability and Flexibility

**DOI:** 10.1371/journal.pcbi.1004331

**Published:** 2015-06-12

**Authors:** Kai Ueltzhöffer, Diana J. N. Armbruster-Genç, Christian J. Fiebach

**Affiliations:** 1 Department of Psychology, Goethe University Frankfurt, Frankfurt am Main, Germany; 2 Bernstein Center for Computational Neuroscience, Heidelberg University, Mannheim, Germany; 3 Department of Neuroradiology, Heidelberg University, Im Neuenheimer Feld, Heidelberg, Germany; 4 Donders Institute for Brain, Cognition and Behaviour, Radboud University, Nijmegen, The Netherlands; 5 IDeA Center for Individual Development and Adaptive Education, Frankfurt am Main, Germany

## Abstract

Cognitive stability and flexibility are core functions in the successful pursuit of behavioral goals. While there is evidence for a common frontoparietal network underlying both functions and for a key role of dopamine in the modulation of flexible versus stable behavior, the exact neurocomputational mechanisms underlying those executive functions and their adaptation to environmental demands are still unclear. In this work we study the neurocomputational mechanisms underlying cue based task switching (flexibility) and distractor inhibition (stability) in a paradigm specifically designed to probe both functions. We develop a physiologically plausible, explicit model of neural networks that maintain the currently active task rule in working memory and implement the decision process. We simplify the four-choice decision network to a nonlinear drift-diffusion process that we canonically derive from a generic winner-take-all network model. By fitting our model to the behavioral data of individual subjects, we can reproduce their full behavior in terms of decisions and reaction time distributions in baseline as well as distractor inhibition and switch conditions. Furthermore, we predict the individual hemodynamic response timecourse of the rule-representing network and localize it to a frontoparietal network including the inferior frontal junction area and the intraparietal sulcus, using functional magnetic resonance imaging. This refines the understanding of task-switch-related frontoparietal brain activity as reflecting attractor-like working memory representations of task rules. Finally, we estimate the subject-specific stability of the rule-representing attractor states in terms of the minimal action associated with a transition between different rule states in the phase-space of the fitted models. This stability measure correlates with switching-specific thalamocorticostriatal activation, i.e., with a system associated with flexible working memory updating and dopaminergic modulation of cognitive flexibility. These results show that stochastic dynamical systems can implement the basic computations underlying cognitive stability and flexibility and explain neurobiological bases of individual differences.

## Author Summary

In this work we develop a neurophysiologically inspired dynamical model that is capable of solving a complex behavioral task testing cognitive stability and flexibility. We can individually fit the behavior of each of 20 human subjects that conducted this stability-flexibility task during functional magnetic resonance imaging (fMRI). The physiological nature of our model allows us to estimate the energy consumption of the rule-representing module, which we use to predict the hemodynamic fMRI response. Through this model-based prediction, we localize the rule module to a frontoparietal network known to be required for cognitive stability and flexibility. In this way we both validate our model, which is based on noisy attractor dynamics, and specify the computational role of a cortical network that is well-established in human neuroimaging research. Additionally, we quantify the individual stability of the rule-representing states and relate this stability to individual differences in energy consumption during task switching versus distractor inhibition. Hereby we show that the activation of a thalamocorticostriatal network involved in the dopaminergic modulation of cognitive stability is modulated by the model-derived stability of the frontoparietal rule-representing network. Altogether, we show that noisy dynamic systems are likely to implement the basic computations underlying cognitive stability and flexibility.

## Introduction

The successful pursuit of behavioral goals often requires the stable maintenance of behavioral plans even in the face of distracting influences from the environment. Equally important, however, is the ability to flexibly adapt behavior to changing environmental demands. These two abilities are often described as cognitive flexibility and stability and are conceptualized as component processes of the executive control of behavior [1,2]. Cognitive stability is often operationalized in delayed response tasks [3,4], in which a stimulus has to be remembered for a short time span (maintenance period) after its presentation, before a decision based on the stimulus has to be made. To test the resistance of the working memory representation, this task is often combined with the presentation of distracting stimuli during the maintenance period [5–7]. Cognitive flexibility can be tested in terms of reversal learning, such as in the Wisconsin Card Sorting Test [8,9], in terms of cue based switching of attention to a different stimulus or stimulus dimension within a single task [10,11], or in terms of cue based task switching [12–15] in which different tasks have to be executed on the same stimulus material.

**Fig 1:**
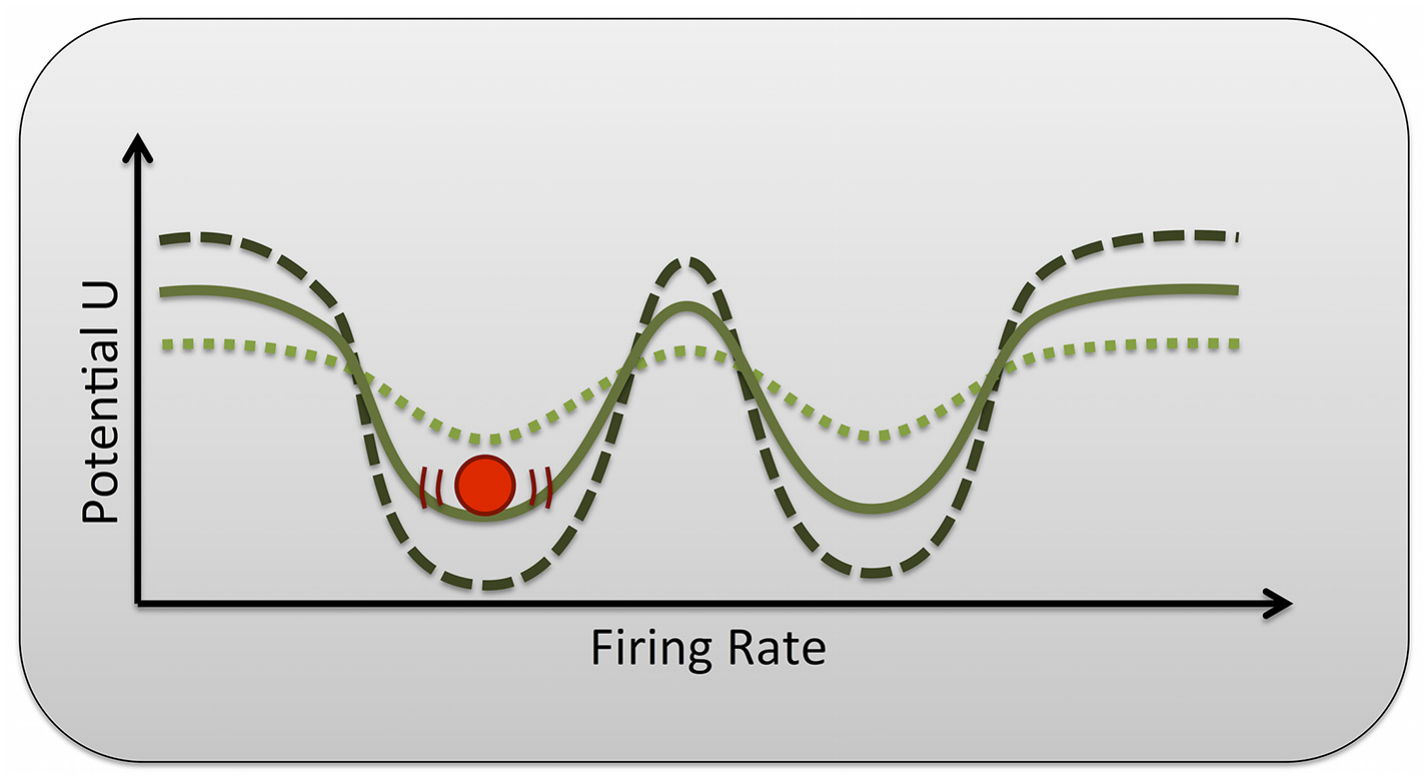
Conceptualized potential landscape. Attractors can be visualized as basins in a potential landscape. The depth of the basin indicates the stability of the associated attractor, i.e., the amount of external forcing or noise that is needed to change the state of the system (here schematically represented by a red ball). Here we show a hypothetical one-dimensional potential landscape of a quite flexible system (dotted line) where only a small potential barrier separates two neighboring basins of attraction, a very stable but less flexible system (dashed line), and an intermediate system (solid line).

Interestingly, even though cognitive mechanisms underlying the stability and flexibility of behavior have typically been examined independently from each other, there is accumulating evidence that these functions share overlapping functional networks [10] and that they are antagonistically modulated by dopamine in healthy subjects [16,17]. Both of this is consistent with computational models of the modulation of working memory maintenance in the prefrontal cortex [18,19]. In terms of these detailed biophysical models, working memory representations can be conceptualized using a potential landscape, as shown in Fig 1. Here different memory items correspond to different minima, so called attractors, of the landscape. To change the content of working memory, the system has to be forced over the ridge separating the currently active from a new memory representation. Thus, while an increase in the depth of the basins of attraction stabilizes the currently active memory, i.e. increases its resistance to inherent noise processes or external distractors, it also increases the internal forcing required to deliberately move the system from one attractor (i.e., working memory or task state) to another.

Although the theoretical idea that attractor states in prefrontal neuronal networks underlie active working memory representations can qualitatively explain the action of prefrontal dopamine on working memory performance [18,19] and was also able to explain antagonistic effects in cognitive stability and flexibility in our own work in humans [12], actual measurements of attractor-like ensemble activity in vivo are still sparse [20,21]. Using data from multiple single unit activity recordings, neural attractor dynamics were directly observed in rats solving a working memory task [21]. Since this study relied on high-resolution multiple single unit recordings, which are hardly feasible in humans, up to now there has been no direct evidence for the actual presence of attractor states supporting working memory in humans. Accordingly, there has also been no direct quantitative link showing that the stability of attractor states is indeed a relevant dynamical quantity of the neural dynamics underlying cognitive stability and flexibility.

**Fig 2:**
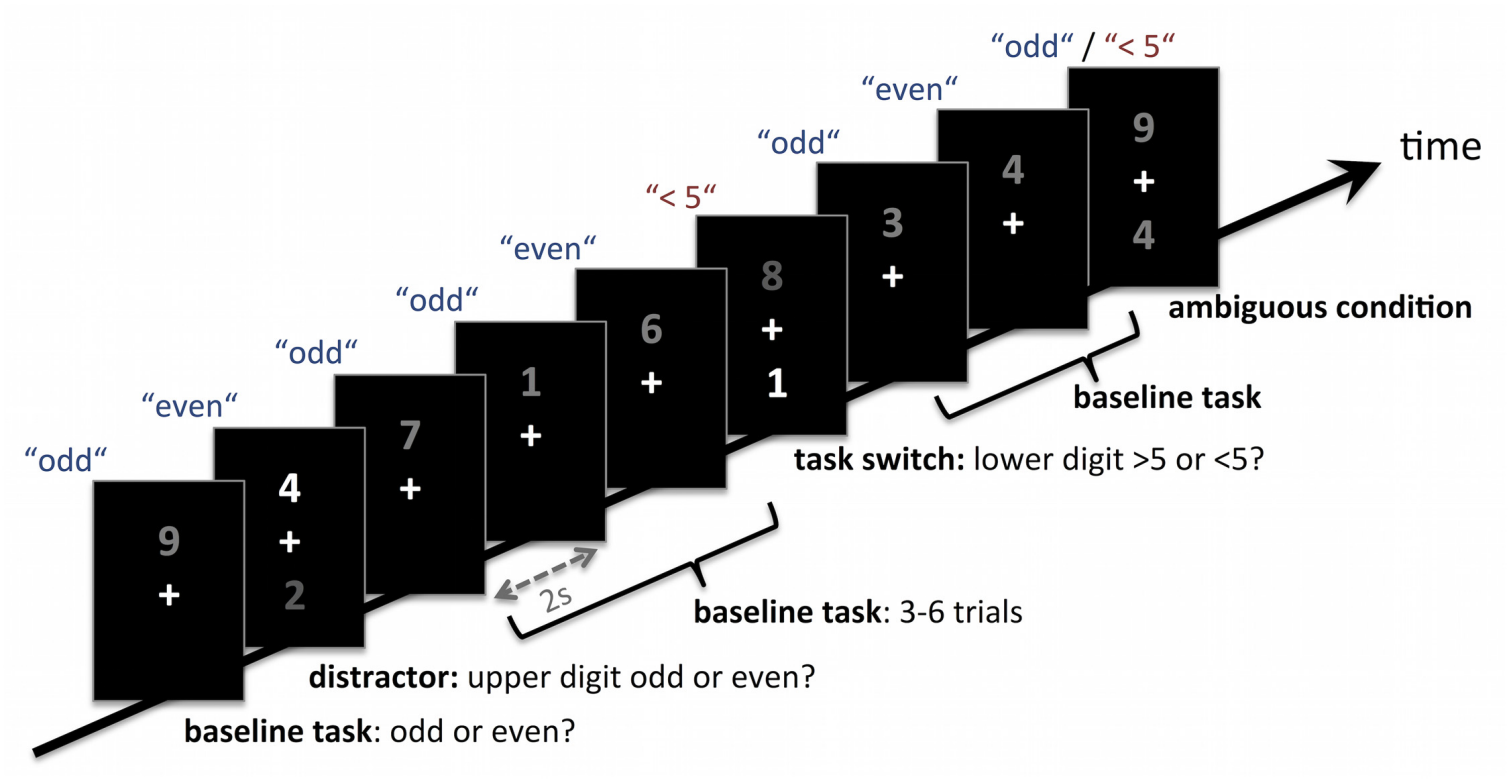
Schematic illustration of the task. 80% of the trials required a response to a single digit presented above the fixation cross, i.e., to decide whether it is odd or even (baseline task; parity judgment). In 20% of the trials, a second digit appeared below the fixation cross. Subjects had to ignore this bottom digit if it was darker then the upper digit and continue to respond to the upper digit, indicating its parity (distractor condition). If the bottom digit was brighter than the upper digit, subjects had to switch the task rule, now indicating if the target was greater than or less than five (magnitude judgment), and apply that rule to the bottom digit (task switch).

In this paper, we develop a stochastic dynamical model derived from physiologically plausible network models of working memory and decision-making [22–26]. Our model can quantitatively fit the behavior of a sample of 20 subjects in a combined task switching and distractor inhibition paradigm [12], which is detailed in Fig 2 and in the Methods section. We use the subject-specific fitted models to predict the individual functional magnetic resonance imaging (fMRI) blood oxygenation level dependent (BOLD) signal timecourses of the modeled network module which implements the working memory maintenance of the currently active task rule. This rule maintenance module is found to map to a functional network consisting of the left inferior frontal junction area (IFJ) and regions within the left intraparietal sulcus (IPS), a network known to be crucially involved in the cognitive control of behavior, including distractor-inhibition and domain-general task-switching [27–29]. Furthermore, we estimate the individual attractor stability of the rule maintenance module from the fitted models and use it to predict the amount of brain activation that subjects need to invest when flexibly switching between tasks. We find that subjects with more stable rule representation networks (i.e., deeper attractors) require during task switching the increased activation of a thalamocorticostriatal network, which is associated with the updating of information in working memory [17,30–32] and dopaminergic modulation of cognitive flexibility [17,33].

In this way we show that attractor states of the IFJ-IPS network can implement the active maintenance of task rules in working memory. Additionally, the stability of these representations modulates the activation of a thalamocorticostriatal updating network during situations requiring cognitive flexibility. This explicitly shows the crucial role of the stability of neural attractor states for cognitive stability and flexibility.

## Results

### Physiologically Plausible Model of a Flexibility-Stability Paradigm

We adapted a network model of task switching [34] to successfully perform a distractor inhibition and task switching paradigm that we have previously developed to probe both cognitive stability and flexibility [12], outlined in Fig 2 and in the Methods section. In brief, participants had to respond fast and accurately by button presses to digits between 1 and 9 (excluding 5) that were presented in different shades of gray against a black background. In 240 of 300 trials, only one digit was shown above the fixation cross using a constant, medium gray value. Participants had to decide whether this digit was odd or even and responded with the index/middle finger of the right hand (baseline condition). For the remaining 20% of trials, which were randomly intermixed between the baseline trials, two digits appeared on the screen, i.e., one above and one below the fixation cross (Fig 2). In this case, the relative brightnesses of the two digits indicated which rule had to be applied: If the upper digit was brighter than the lower digit, participants had to ignore the lower digit (distractor inhibition condition; 20 trials). In this condition, participants were instructed to continue using the odd/even decision rule applied to the upper digit, and to ignore the distracting digit below the fixation cross. However, in the task switch condition (20 trials), the lower digit was brighter than the upper digit, which signaled participants to switch from the upper to the lower digit and to decide whether it was smaller or larger than 5, using the index/middle finger of their other, i.e., left hand. A direct mapping of all four possible decisions (odd, even, >5, <5) to individual fingers allowed us to explicitly infer which task rule was applied on each individual trial. The mapping of rules to hands was counterbalanced across subjects. Finally, the task involved further 7% of ambiguous cue trials in which participants could or couldn't switch. This condition is not considered in the present modeling work.

**Fig 3:**
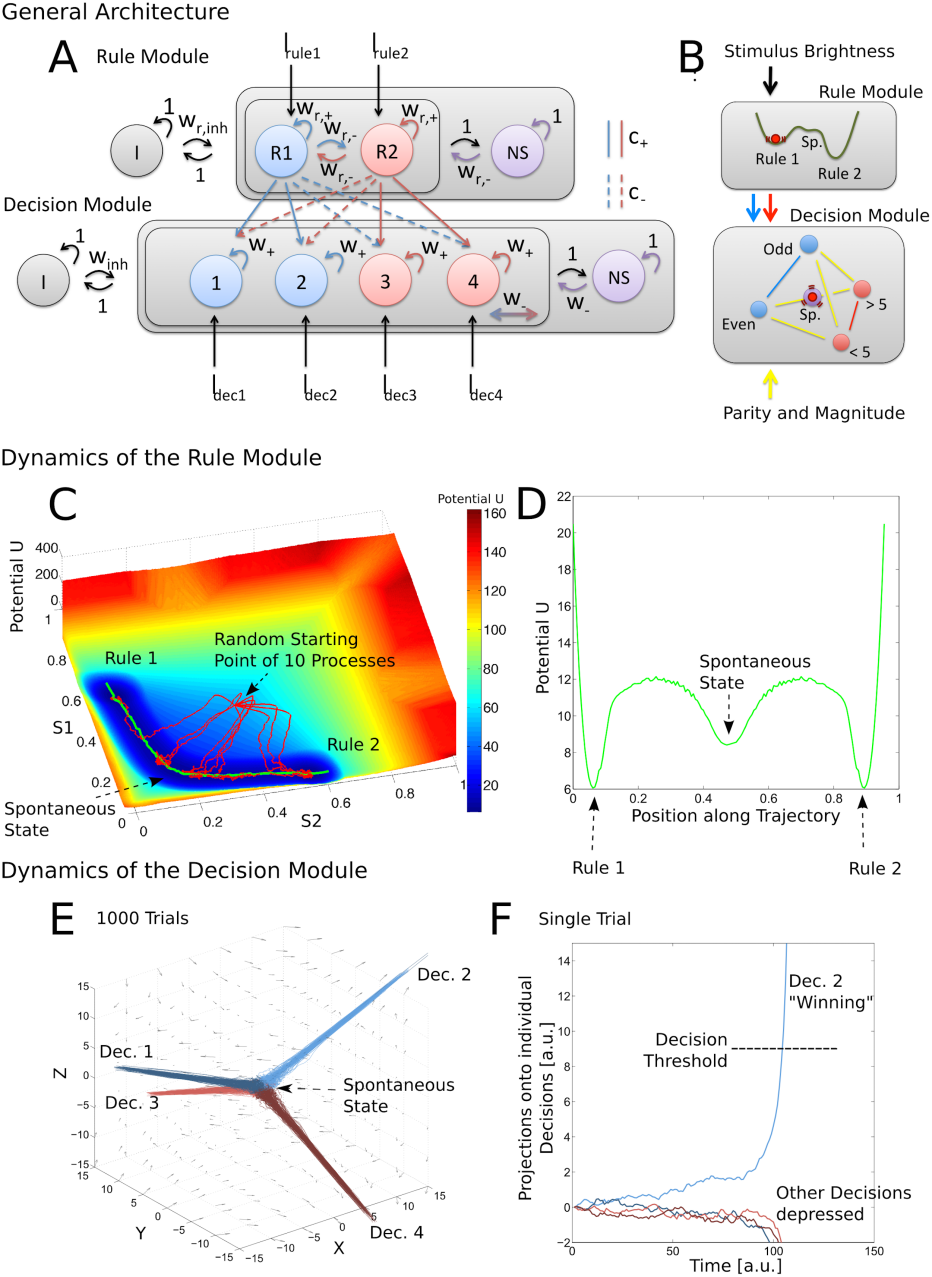
Model architecture and dynamics. (A) Original architecture of the network: Rules are represented by two selective populations (R1, R2) embedded in a pool of nonselective excitatory (NS) and inhibitory (I) neurons. Decisions are implemented by the same architecture of four decision selective populations. The interplay of strong local excitation and global inhibition creates winner-take-all dynamics in both modules, leading to a stable state of globally low spiking activity and stable states corresponding to the high activity of a single selective population. (B) Schematic of the stochastic dynamics of reduced system: The rule module is reduced two a two dimensional dynamical system with three stable attracting states: A spontaneous low-activity state and two rule-selective states of high activity. The decision module is reduced to a three-dimensional nonlinear drift-diffusion process, starting at the center of a tetrahedron, where each corner represents one of the four possible choice alternatives. A drift towards one of the corners is created by a combination of direct stimulus input, which favors one of the yellow edges, and biasing input from the selective populations of the rule module, which favors either the red or the blue edge of the tetrahedron. (C) Dynamics of the rule module: The rule module is reduced to a two-dimensional system whose phase space is represented by the synaptic gating variables (S1, S2) of the two rule selective populations. The model is set up in a way in which the two rule representing states as well as the spontaneous state are stable. Trajectories (red) started at an arbitrary point explore the phase space, due to the noise, but tend to stay close to one of the attractors, due to the deterministic dynamics. (D) Slice through a potential landscape of the rule module. This cross-section following a path connecting the two rule attractors via the spontaneous state illustrates the potential barrier that has to be overcome for transitioning from one state to another. Theoretically, all two-dimensional paths are possible to transition from the rule 1 to the rule 2 state. However, due to its dynamical properties, the system stays in a valley that connects the two rule attractors via the basin of attraction of the spontaneous state. The cross-sections along those paths resemble closely the one-dimensional potential sketches often found in the literature discussing the possible effects of attractor stability on cognition [18,19]; compare Fig 1. (E) Dynamics of the decision module: The figure shows the evolution of a spherical volume in the three dimensional phase space centered around the origin, which corresponds to the spontaneous state of the decision network. The trajectories rapidly converge to one of the four directions corresponding to the winner-take-all states of one of the decision selective populations. Due to the symmetry of the system, these directions form the corners of a symmetrical tetrahedron. (F) Threshold independence of decision module: Shown are the projections of a single three-dimensional decision process onto the four directions corresponding to the increase of a single selective population and the simultaneous decrease in all others. Due to the nonlinear dynamics, the trajectory rapidly converges into one of these directions and then diverges to infinity within finite time. This yields decision times that are insensitive to the exact value of the threshold, as long as it is placed far enough from the spontaneous state.

The model architecture is illustrated in Fig 3A and the dynamics of the model are sketched in Fig 3B. The network consists of a rule maintenance module, which represents the currently active task rule (out of two possible tasks; see above and Fig 2), and a decision module, which integrates top-down bias exerted by the selective populations of the rule module with bottom up input encoding the presented stimuli to generate a behavioral output (out of four possible responses). The rule module consists of two rule-selective populations (R1, R2) of excitatory neurons that feature strong recurrent excitatory connections within and weak excitatory connections in between the selective populations [22]. The embedding into a shared pool of inhibitory interneurons and non-selective excitatory neurons creates a global competition leading to winner-take-all dynamics. When one of the populations is driven across a threshold by external stimulation, it can sustain its high activity state by means of recurrent excitation even after the stimulus has ended, while simultaneously depressing the second selective population by driving the shared inhibitory neurons. This system can exhibit three possible stable states: a spontaneous state in which all populations fire with a low, background rate, as well as one of two high activity states in which one of the selective populations fires with high average activity, sustaining its own firing and depressing the other selective population via shared inhibition. This fundamental architecture is consistent with physiological features of the neocortex [35–38].

We implemented the dynamics of the rule maintenance module using a reduced firing-rate model [25,39]. The state space of the system is spanned by the synaptic gating variables S1 and S2 of the rule 1 and rule 2 selective populations, respectively, describing the average synaptic activity within the population. The resulting stochastic dynamical system has three stable fix-point attractors: two high-activity states corresponding to the two possible rules and a low-activity spontaneous state, as shown in Fig 3C. The heuristic reduction developed in [25] conserves the effects of the physiological NMDA, GABA, and AMPA scaling parameters. In contrast to the implementation in [25], we model finite size fluctuations using an Ornstein-Uhlenbeck process with diffusion parameter σ_rule_, which is directly added to the dynamics of the synaptic gating variables (cf. Methods). This allows us to visualize and quantify the effective potential landscape of the rule module. The influence of physiological parameters on the landscape and the dynamics of the rule module will be discussed below.

The decision module shares the same underlying architecture, using four decision-selective excitatory populations with strong recurrent connections and global pools of inhibitory and non-selective excitatory neurons. Due to the recurrent excitation and shared inhibition, a single decision-selective population can sustain its activity while inhibiting the other selective populations, again creating winner-take-all network dynamics. We implemented the dynamics of the decision module by generalizing a nonlinear drift-diffusion equation, which was shown to capture the dynamics of a decision network with two selective populations and firing rate dynamics similar to our rule module [26], to four decision selective populations. This additional step of reduction allowed us to formulate the dynamics of the decision module as three-dimensional, nonlinear drift-diffusion process.

The resulting dynamics automatically capture the winner-take-all dynamics of the underlying neural attractor network, as shown in Fig 3E: Processes that are initialized close to the origin of the coordinate system, which corresponds to the spontaneous state of the decision network, rapidly converge to one of four directions representing the possible winner-take-all network states. This allows very fast, efficient, and stable simulation of thousands of behavioral trials. Of course, this reduction comes at the expense of a degeneracy of the effective parameters of the decision module with respect to physiological parameters, which can therefore not be recovered anymore from our fits. Additionally, we loose the stability of high-activity states in the decision module, which prevents us from making predictions about the energy consumption of the decision module. But since the present work focuses on the role of the working memory representation of the currently active task rule in the rule module, and as the decision model is—in combination with the rule module dynamics—able to quantitatively fit our behavioral data (see below), we decided that the improvements in speed, convergence, and stability outweigh the additional abstraction introduced by this comparatively simple model of the decision making process. For a detailed discussion of the dynamics of the decision module and its derivation from a generic rate model see the Results section on "Physiologically Derived Nonlinear Drift-Diffusion Model for Four-Choice Tasks" and the Methods section on "Dynamics of the Decision Module" below.

Behavioral data can be generated using the transition of the current system state of the decision module (Fig 3B bottom panel, red ball) from the spontaneous state (purple sphere in Fig 3B, origin in Fig 3E) to one of the four high-activity states (light red and blue spheres at the corners of the tetrahedron in Fig 3B, corresponding to the divergence of the process to one of the four directions in Fig 3E, which represent the increase in the activity of one decision selective population with the simultaneous decrease in the other decision selective populations). By biasing the decision-selective populations using top-down inputs from the rule selective populations (solid red/blue lines in Fig 3A) and bottom-up inputs encoding the presented stimuli (Fig 3A bottom, black solid lines), realistic reaction time distributions and decision probabilities can be generated [23,24,40–42].

An individual task trial is simulated by initializing the rule module to the high activity state of the rule 1 population. This rule has to be applied in the ongoing baseline condition and the distractor condition of the behavioral task, which together represent 87% of the trials. Therefore the high activity state of this rule is a plausible initial state of the network. The relative brightness of the presented stimuli, which serves as task cue for rule 1 vs. rule 2, is encoded by applying excitatory inputs of different strengths to the corresponding selective populations of the rule module. The simultaneously presented stimuli themselves are encoded by excitatory inputs to the decision-selective populations, which are described in detail in the Methods section “Simulation of Behavioral Data”. For the ongoing baseline task, this involves input only to decision selective pools 1 and 2 of the decision module, corresponding to the odd and even decisions, as only one stimulus is presented (compare Fig 2, baseline condition). A stronger input is applied to the pool representing the parity of the presented stimulus. During the task switching and distractor inhibition trials, two stimuli were presented. This is implemented as excitatory input to decision selective pools 1–4, with stronger inputs to the selective populations representing the parity and magnitude of the presented stimuli. This setup produces different dynamics depending on condition, that are schematically visualized in Fig 3B: In the baseline condition, the unambiguous bottom-up input creates a direct drift of the system state (red ball) to the even or odd decision (blue spheres in Fig 3B). In the distractor and switch conditions, where two stimuli are presented, the perceptual input produces a drift towards one of the yellow edges. In order to solve the task correctly, the decision module now needs an additional forcing into one of the task representing directions (red and blue lines in Fig 3B) by a top-down bias from the rule module. This top-down influence from the rule module onto the decision module is accomplished by forwarding the current firing rates *r*_1_ and *r*_2_ of the rule-selective populations as drift in the directions equivalent to increased activity in the corresponding decision selective populations with the feed-forward weight *c*_+_, as shown in Fig 3A. Depending on the currently active task rule, this top-down input from the rule module drives the decision process to either the red or the blue edge in Fig 3B. Thus, by the combination of top-down inputs from the rule module with the bottom-up stimulus inputs, the population corresponding to the specific decision that is consistent both with the currently active task rule and the presented stimulus features gains the highest excitatory inputs. These drifts, together with the inherent noise in the network, lead to a distribution of decisions in which the correct decision is taken with highest probability.

### Physiologically Derived Nonlinear Drift-Diffusion Model for Four-Choice Tasks

**Fig 4:**
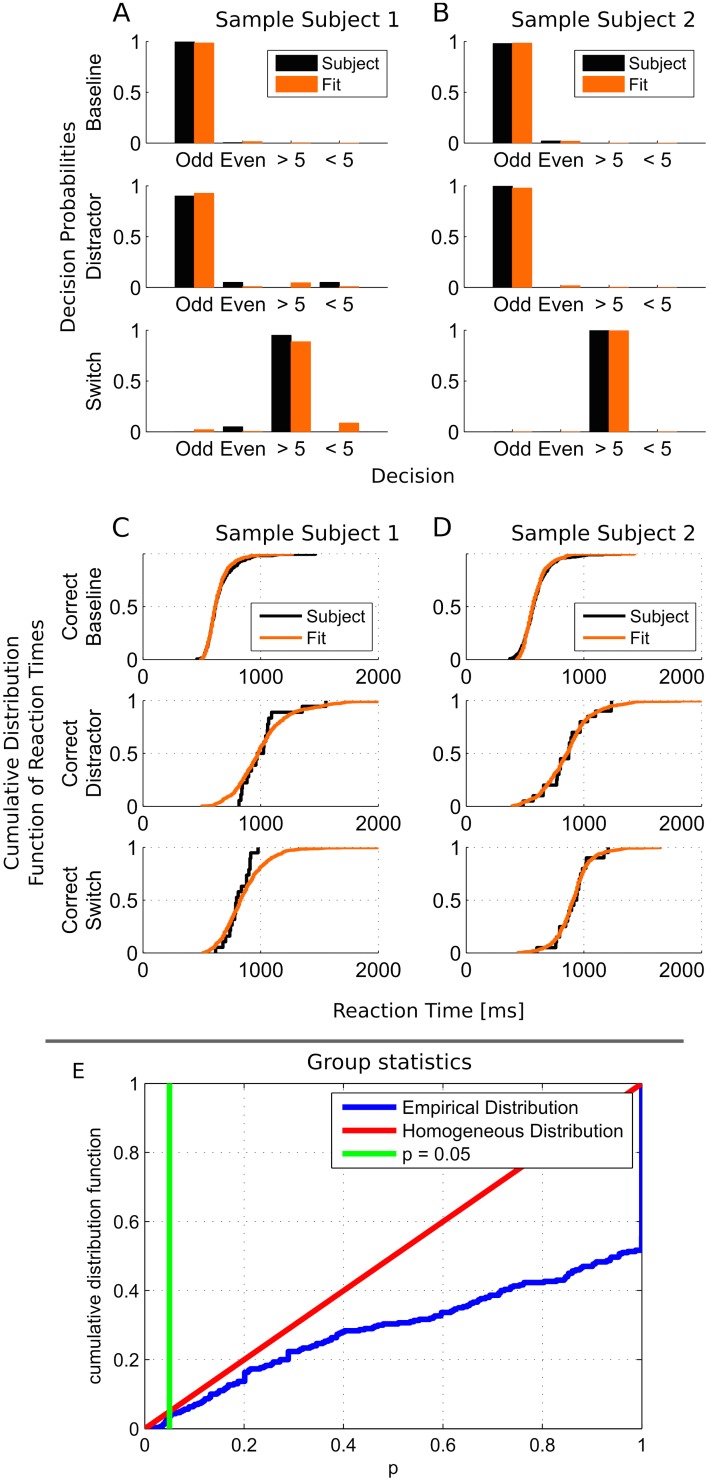
Reproduction of behavioral data. (A/B) Decision probabilities assuming the presentation of a stimulus with odd parity in the baseline condition and a stimulus with odd parity above and a stimulus with magnitude greater than 5 below the fixation cross for the distractor and switch condition for two representative subjects (Subjects 26 and 9). Empirical data for each subject is drawn in black, simulated data generated from the fitted models are drawn in orange. Despite the overall very good quality of the fit, one can note the comparatively better fit in the baseline condition. This is due to the higher contribution of the associated multinomial factor to the Bayesian posterior distribution, which strongly depends on the number of experimental trials. (C/D) Cumulative distribution function of reaction times for correct baseline, distractor, and switch trials for the same subjects (empirical data: black; simulated/fitted data: orange). Note again the relatively low resolution of the empirical cumulative density functions in the distractor and switch conditions, due to the relatively low number of trials. (E) Group statistics describing the general goodness of fit across all subjects. Cumulative distribution function of p-values obtained by individual exact multinomial tests and two-sample Kolmogorov-Smirnov-tests of the Null hypothesis that the behavioral data was drawn from distributions whose parameters were derived from the corresponding fitted model (blue). For comparison, a homogeneous distribution on [0,1] is shown in red. The uncorrected significance threshold (p = .05) is shown as solid green line. Note that only 3% of the performed tests fell under the significance threshold. Almost half of the tests reached p = 1.

We reduced the decision module to the diffusion model outline above by generalizing an approach developed by Roxin and Ledberg [26]. We start from a generalized rate model of the population dynamics of the decision module. This expresses the dynamics of the four selective populations and the shared inhibitory population in the form of five coupled ordinary differential equations (cf. Methods). We reduce the dynamics close to the spontaneous state at its bifurcation point, i.e. when the spontaneous state is just losing its stability. This means that the matrix describing the linearized dynamics at the spontaneous state is just becoming singular, therefore featuring only negative and zero eigenvalues. This reduces the dimensionality, since the system can only evolve freely in the directions corresponding to the zero eigenvalues, while the dynamics in the other directions—due to the corresponding negative eigenvalues—decay quickly, thereby enslaving the other dimensions to follow those dynamics. This produces a three dimensional phase space in which the directions corresponding to the increase of activity in one selective population and the simultaneous decrease of activity in the other selective populations form a symmetrical tetrahedron, which is illustrated in Fig 3E. The nonlinearity of the dynamical equations automatically incorporates the winner-take-all dynamics of the underlying network model. This leads to the fast convergence of the trajectories to the directions corresponding to the winner-take-all states of a single population. The nonlinearities also lead to a divergence of the solution in finite time (Fig 3F), which makes the decision module insensitive to the exact placement of a decision threshold, as long as it is far enough away from the spontaneous state. In this way, the method naturally generalizes drift-diffusion models—that are often used to model reaction time distributions from two-choice tasks—to multiple choices [43,44]. By combining this module with drift parameters corresponding to top-down input from the rule module and bottom-up input encoding of the stimuli, we were able to fit the behavioral statistics of all individual subjects (Fig 4, cf. Methods). It is notable that the structure of the three-dimensional decision space and the embedding of the choices in the form of a symmetrical tetrahedron emerged naturally from the reduction of a generalized system of the type introduced in [25].

### Reproduction of Behavioral Data

We implemented the reduced stochastic attractor and nonlinear drift-diffusion model using the C++ programming language together with the NVIDIA CUDA toolkit for parallel computing on graphics hardware [45]. In this way we were able to simulate several thousands of trials on consumer graphics hardware (NVIDIA GeForce GTX Titan) in parallel in less than 100 ms. This allowed us to directly sample the Bayesian posterior distribution of the model parameters given the behavioral data of an individual subject using a Markov-Chain-Monte-Carlo (MCMC) sampling scheme. A sample of the marginalized single-parameter distributions generated is shown in S1 Fig (cf. also Methods; full codes at https://sourceforge.net/projects/mcmc-mp/). Our model was able to accurately reproduce the behavior of 20 individual subjects in the baseline, distractor inhibition, and task switch conditions. Two representative subjects are shown in Fig 4, all subjects are shown in S2 and S3 Figs, and mean statistics for the full sample and the corresponding fits are shown in S4 Fig. To quantify the quality of the fit we used exact multinomial tests on the decision distributions for each condition. In these tests, the rates of the multinomial distributions were given by the relative decision probabilities of the fitted model in the baseline, distractor, and switch condition. We calculated the p value for the Null Hypothesis that the behavioral data of the subjects were drawn from these distributions. Additionally, we used the two-sample Kolmogorov-Smirnov test for each combination of condition and decision to obtain the p value of the Null hypothesis that the simulated and experimental reaction time distributions were drawn from the same underlying probability distribution. The cumulative distribution function of all tests performed is shown in Fig 4E. Using a significance threshold of p > .05, there are hardly any significant differences (only 10 of 300 tests), especially considering that at least six tests were performed for each individual subject (on the decision distributions in the baseline, distractor and switch conditions, and on the reaction time distributions of correct baseline, distractor and switch trials). For almost 50% of all tests performed, the p-value is 1, showing a perfect correspondence of the fitted models and experimental data. This means that the cognitive dynamics in terms of decision probabilities as function of task condition, as well as the full reaction time distributions can be explained by our relatively simple, physiologically plausible model of two modules of neocortex.

### Localization of the Rule Module

**Fig 5:**
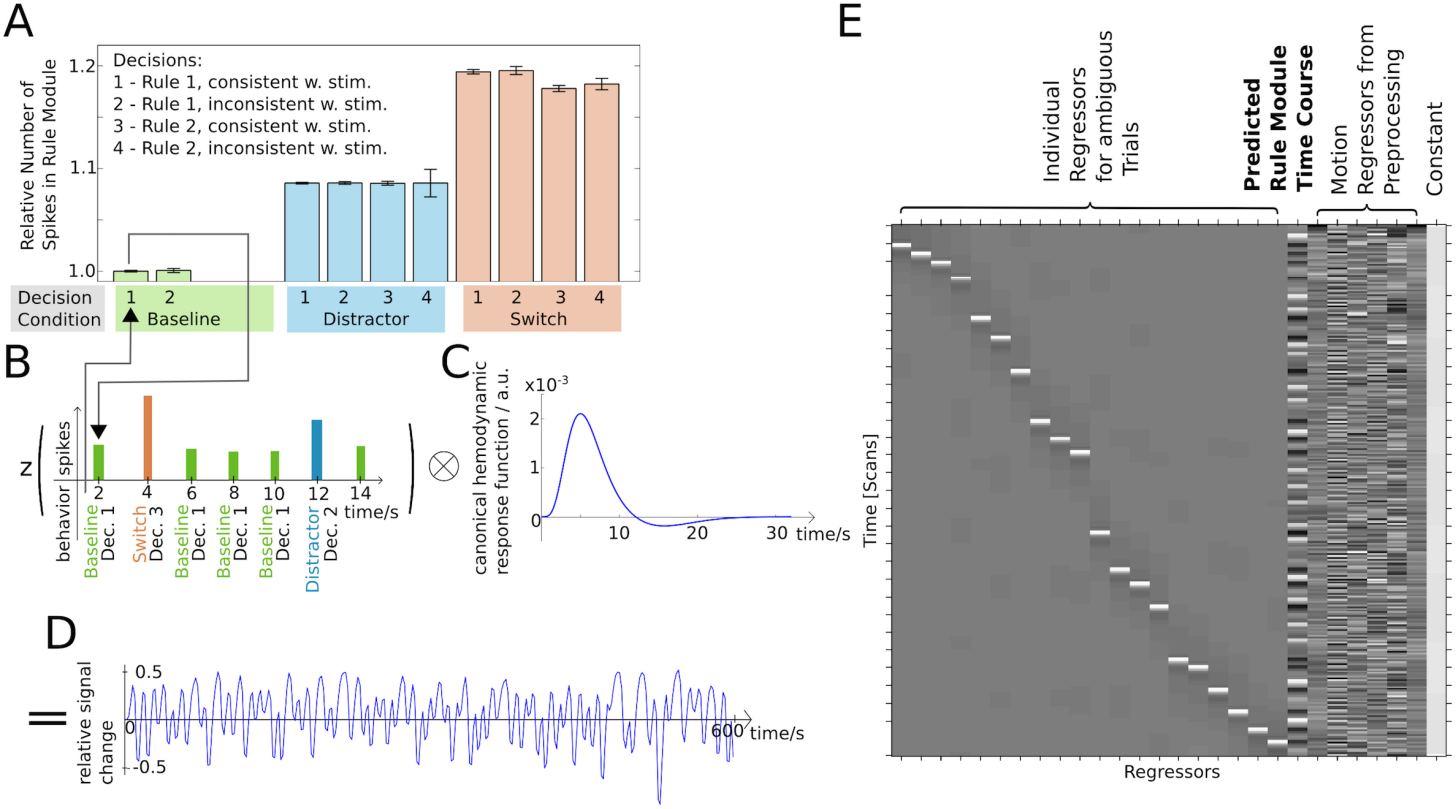
Generation of fMRI BOLD timecourses from fitted models. (A) Simulated energy consumption of the rule module. Simulated trials were sorted by condition and decision taken, and the average integral of the sum of the spiking rates *r*_1_+*r*_2_ over the course of a trial was calculated. This estimate of the energy consumption was normalized relative to the average spiking rate integral of a correct baseline trial. (B) Depending on the condition and decision recorded in the individual participants’ behavioral logs, the corresponding energy estimates were placed on a timeline. The resulting time course was z-scored. (C) The normalized timecourse estimating the neural energy consumption was then convolved with a canonical hemodynamic response function (as derived from the SPM8 software package), resulting in (D) a predictor timeseries representing the activation of the rule module over the course of the experiment, individually for each participant. (E) This regressor was entered into a multi-univariate GLM of the functional MRI data, together with regressors for each individual ambiguous trial and with six motion regressors derived from the coregistration of the functional MRI volumes (see Methods section, “Generation and Localization of fMRI Timeseries from Fitted Models”).

Given that we had functional MRI data during performance of the cognitive stability/flexibility task available for all 20 participants (cf. [12]), we reasoned that examining the localization of the simulated rule module might serve as validation for the model presented here. To this end, we used the fitted models together with the subject-specific behavioral log files and a canonical hemodynamic response function (SPM8, http://www.fil.ion.ucl.ac.uk/spm/software/spm8/, typically used for standard fMRI analyses) to predict the individual BOLD time course of the rule module for each of the 20 subjects, as shown in Fig 5: We calculated the average integral of the sum of the spiking rates *r*_1_+*r*_2_ of the two rule selective populations over the course of a trial as function of condition and decision. As shown in Fig 5A exemplarily, the main difference in the number of spikes during a trial is mediated by the condition. This is due to the fact that in our task the task-cue is encoded in the relative brightness of the two stimuli, with a brighter stimulus above the fixation cross indicating the application of the parity rule and a brighter stimulus below the fixation cross indicating the use of the magnitude rule, as illustrated in Fig 2. This is implemented by corresponding excitatory inputs to the selective populations of the rule module: During the baseline trials, only one stimulus is shown above the fixation cross, i.e., the position associated with the parity rule. During the simulation of these baseline trials, we only apply a forcing to the rule 1 (parity rule) population. In the distractor condition, there is again a bright stimulus above the fixation cross, but additionally there is a darker, distracting stimulus below the fixation cross. This is simulated by applying a strong external input to the rule 1 (parity) and a correspondingly weaker input to the rule 2 (magnitude) population. This additional input leads to an increase in the global activity of the rule module, which manifests in an increase in the spiking-rate integral in the module.

During switch trials, the relative brightness of the stimuli is the other way round: There is now a dark stimulus above the fixation cross, and a bright stimulus below it, which has to be responded to by applying the other task rule, i.e., the magnitude rule. Since the rule module is always initialized in the high activity state of rule 1, which has to be applied in 87% of the trials and which therefore is a plausible initial state of the system, now there has to be a switch in the rule module from the high activity attractor state of rule 1 to the high activity state of rule 2. This is necessary for the decision module to be able to make a choice based on the magnitude of the stimulus, by integrating the top down rule input with the bottom up stimulus inputs, as shown in Fig 3A and 3B. This switch in turn requires a larger total forcing, which in turn yields an even more pronounced increase in the activity of the module, resulting in a substantially higher average total spike count. To be able to fit individual performance well and to be sure that the resulting pattern of activation is not due to a certain, fixed, and arbitrary input pattern, we fitted the value of the excitatory inputs encoding the relative brightness of the stimuli, and therefore the task cue, for each individual subject, together with the physiological parameters of the rule module, the parameters of the decision module, and the inputs encoding the stimuli, as described in detail in the Methods section (cf. "Simulation of Behavioral Data" and "Fitted Parameter Set"). Using the behavioral log files, which recorded the conditions, stimuli presented, and decisions made by the individual subjects, we can use those average spike counts to estimate the neural energy consumption of a hypothetical region implementing this network, as shown in Fig 5B. By z-scoring and convolving this time course with a canonical hemodynamic response function (SPM8, Fig 5C), we can generate a prediction for the BOLD timecourse of such a hypothetical region (Fig 5D).

**Fig 6:**
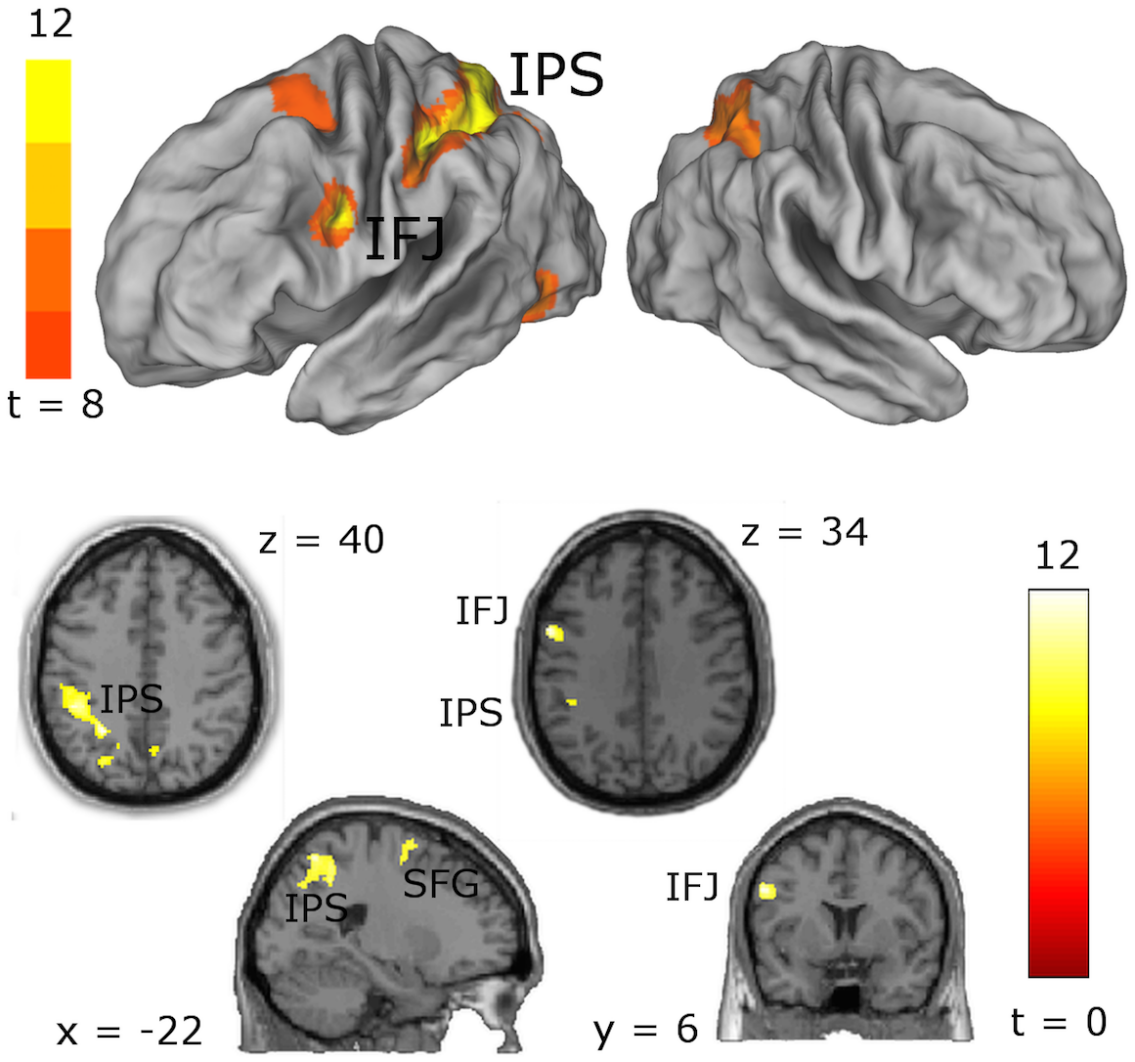
Localization of the rule module. Using the predicted BOLD time courses for the modeled rule module, we were able to localize it to the left inferior frontal junction (IFJ) and the left intraparietal sulcus (IPS), both known to be crucially involved in task switching and distractor inhibition. Weaker activity is observed in the superior frontal gyrus (SFG). The single-voxel threshold of *p* = 10^–7^ corresponds to *p* = 0.05 Bonferroni corrected for the total number of voxels.

We used a standard voxel-wise general linear model to find regions whose measured BOLD timecourse can be predicted by the subject-specific simulated time course of the rule module. This model included six additional regressors to account for subject movement in the scanner and additional regressors for each individual trial of the ambiguous task condition (that is not considered in the present report). An exemplary design matrix is shown in Fig 5E. Using a very conservative threshold of p < 10^–7^, corresponding to p = 0.05 whole-brain Bonferroni corrected for multiple comparisons for the group-level analysis, we could localize the rule module to a network consisting of the left inferior frontal junction region (IFJ) and the left intraparietal sulcus (IPS). Significant but substantially weaker correlations were furthermore found with clusters in the left superior frontal sulcus (SFS), right IPS, and left occipital cortex, as shown in Fig 6 and Table 1.

**Table 1:** Brain regions correlating with the BOLD time-course predicted by the fitted rule module.

			*MNI coordinates of peak voxel*				
*Brain region*	*BA*	*Hemisphere*	*x*	*y*	*z*	*# voxels* *	*T*_*max*_
inferior frontal junction (IFJ)	9	left	-56	8	34	98	12.12
intraparietal sulcus (IPS)	2/7/19/40	bilateral	-24	54	40	1753	12.17
superior frontal sulcus (SFG)	6	left	-22	2	66	52	8.90
inferior occipital gyrus	37	left	-44	-62	-10	74	9.24

* voxel size = 2x2x2 mm^3^

### Reconstruction of the Potential Landscape of the Rule Module

Using methods adapted from the analysis of stochastic reaction and gene-expression networks [46,47], we were able to recover the individual potential landscape of the rule module, depending on a given parameter set (i.e. the NMDA, GABA, and AMPA scaling parameters, and the diffusion parameter of the Ornstein-Uhlenbeck noise process). As the dynamics of the rule module are not conservative, there exists no potential in the classical sense. However, we can use an analogy to statistical physics: Here, the probability of encountering a thermodynamical system in a state described by the parameter vector $\vec{x}$ is $P=\frac{1}{Z}\text{exp}\left(-\frac{U(\vec{x})}{kT}\right)$. This so called Boltzmann-relation connects the probability density, describing the odds of finding a stochastic dynamical system in a certain state $\vec{x}$, with the potential energy *U* associated with this state. Here *k* is the Boltzmann constant, *T* is the temperature of the system, and *Z* is a normalization constant called the partition function. The finite temperature *T* allows a physical system to occupy states beyond the ones with minimal energy, similar to the noise in the rule module, which is able to spontaneously drive the system around within a basin of attraction or even—but with significantly lower probability—from one basin of attraction to another. This strong dependence of the likelihood $P(\vec{x})$ of occupying a given state $\vec{x}$ on the state's potential energy $U(\vec{x})$ is made explicit in the Boltzmann relation. In this spirit, we calculate the generalized potential landscape *U*(*S*_1_,*S*_2_) of the rule module, by calculating the steady state probability distribution *P*_*ss*_ of the rule module, given a fitted parameter set, and then solving the Boltzmann relation for *U* = −ln*P*_*ss*_.

**Fig 7:**
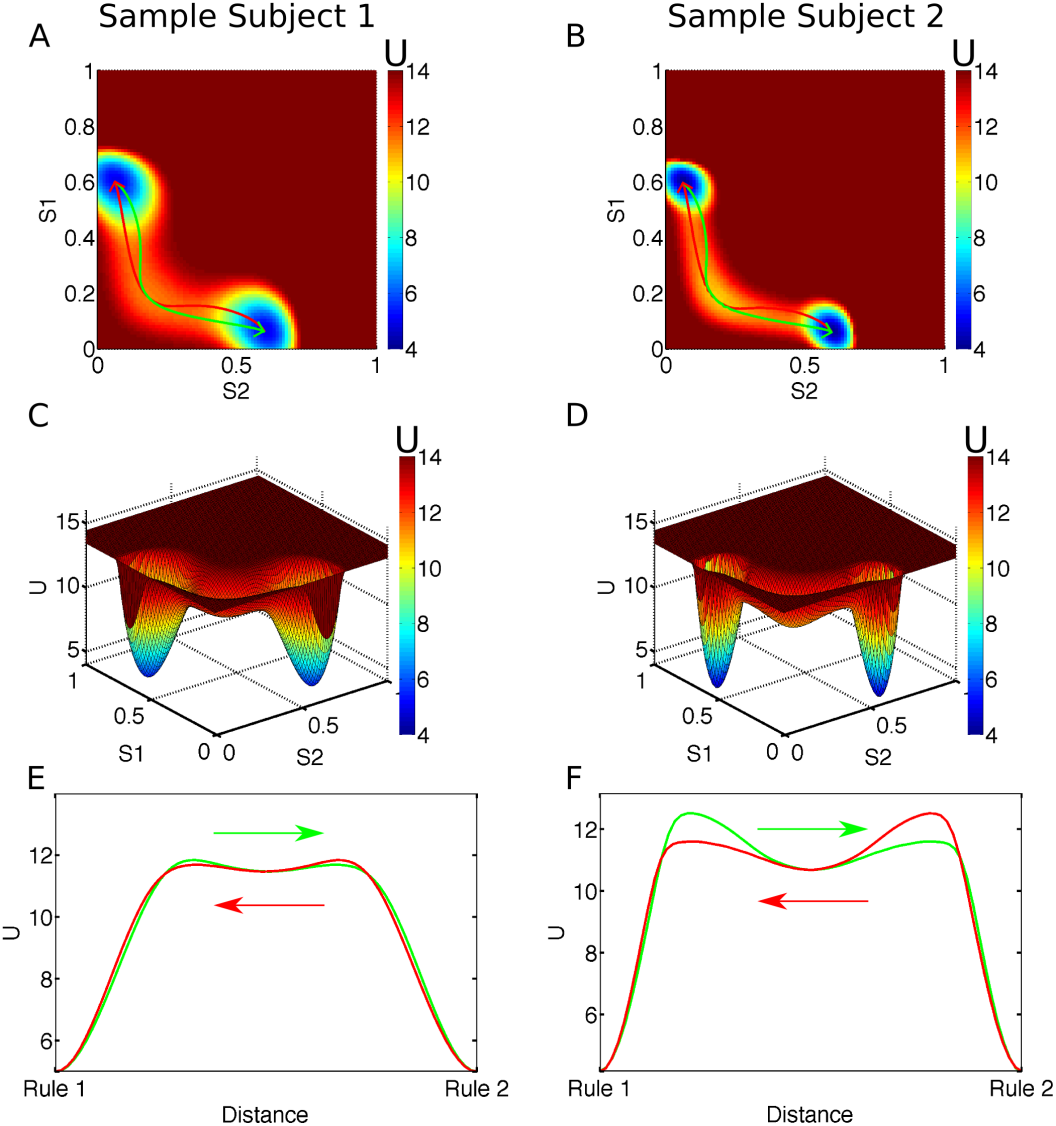
Reconstructed potential landscapes. (A/B) Color map of the reconstructed potential *U* = -ln*P*_*ss*_ on the phase space spanned by the synaptic gating variables (S1, S2) of the rule selective populations for two representative subjects (subjects 26 and 9). The green line indicates the transition from the rule 1 to the rule 2 attractor that minimizes the path-integral action. The red line corresponds to a transition in the opposite direction. (C/D) Surface plot of the reconstructed potential landscapes. Note the deeper and steeper basins of attraction for subject 9. (E/F) Plot of the potential along the transition from the rule 1 to the rule 2 attractor that minimizes the path-integral action (green) and back (red). The individually fitted noise parameters (*σ*_*rule*_) for each subject were scaled by a factor of 10 for easier visualization.

**Fig 8:**
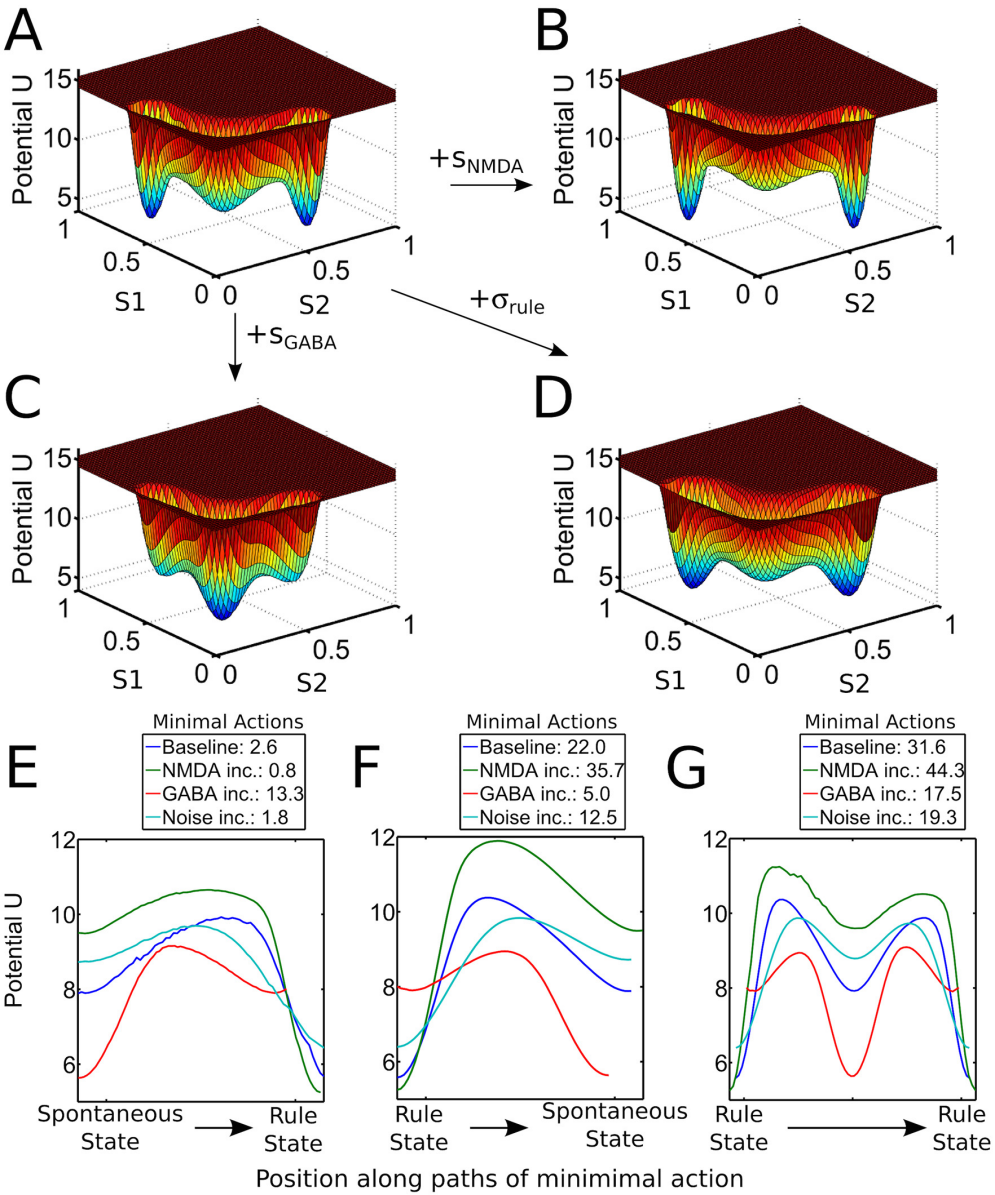
Relationship between physiological parameters and dynamic potential landscape of the rule module. (A) Potential landscape of the rule module on the phase space spanned by the synaptic gating variables (S1, S2) of the two rule-selective populations with standard parameters *s*_*NMDA*_ = 1.0,*s*_*GABA*_ = 1.0,*σ*_*rule*_ = 0.1. (B-D) Changes in the potential landscape of the rule module relative to that standard parameters, i.e., depending on individual increases of (B) *s*_*NMDA*_ = 1.005, (C) *s*_*GABA*_ = 1.005, and (D) *σ*_*rule*_ = 0.15. (E) Potential along the paths corresponding to the minimal action transition from the spontaneous state to a high-activity state for the parameter combinations from A-D. The minimal actions required for the transitions are given in the legend. (F) Potential along the paths corresponding to the minimal action transition from a high-activity state to the spontaneous state for the parameter combinations from A-D. The minimal actions required for the transitions are given in the legend. (G) Potential along the paths corresponding to the minimal action transition between the two high-activity states for the parameter combinations from A-D. The minimal actions required for the transitions are given in the legend.

To calculate this steady state probability distribution, conceptually, one can imagine starting thousands of processes described by the stochastic dynamics of the rule module on random points of the state space spanned by the synaptic gating variables $\vec{S}$, and propagating them for a very long time. Then one can just count the relative number of processes found in each given volume of the state space. Practically, this can be achieved directly by integrating the Fokker-Planck-Equations of the stochastic dynamical system [47]. These equations can be derived from the dynamics of the rule module and directly describe the evolution of probability densities on the phase space. Using these partial differential equations, one can propagate an arbitrary initial probability density until it reaches a steady state. This state does not depend on the initial configuration anymore, but only on the stochastic dynamics of the rule module. Now we can just invert the Boltzmann relation, to define an effective potential *U* = -ln*P*_*ss*_, on the basis of this steady state distribution *P*_*ss*_. The resulting potential landscape is shown for an exemplary parameter set in Fig 3C and using the fitted parameters of two representative subjects in Fig 7. The influence of physiological parameters is visualized in Fig 8 and discussed below (cf. "Shaping of the Attractor Landscape by Physiological Parameters" and Discussion). Due to the dynamics, the system tends to stay close to a valley connecting the rule attractors via the basin of attraction of the spontaneous state. A cross section of the potential landscape along a path from rule 1 to the spontaneous state to rule 2 (green line in Fig 3C; see also Fig 7E and 7F) is shown in Fig 3D.

Due to the nature of their definition, the potential landscapes only capture the steady state probability density on the state space via the Boltzmann relationship *U* = -ln*P*_*ss*_. For the probability distribution to be in a steady state, the divergence of the probability flux has to vanish: $\vec{\nabla }\cdot \vec{J}=0$. While for a conservative system this is solved by $\vec{J}=0$, in a non-conservative system, as it is the case for the rule module, there might still be a non-zero flux, which has to be the curl of an underlying vector potential $\vec{A}$ to fulfill the above condition: $\vec{J}=\vec{\nabla }\times \vec{A}\Rightarrow \vec{\nabla }\cdot \vec{J}=\vec{\nabla }\cdot (\vec{\nabla }\times \vec{A})=0$[47]. The curl-flux, which is not captured by the potential function *U*, leads to the fact that the trajectories for the transition from the rule 1 to the rule 2 state (green trajectories in Fig 7A, 7B, 7E, and 7F) and back (red) are symmetric, but not identical. Physiologically, a transition from the rule 1 to the rule 2 state (green) is initiated by an overproportional increase of activity in the rule 2 population, which leads to the curved shape of the trajectory leaving the initial rule 1 attractor state. Apparently this initial push is required to efficiently transit to the basin of attraction of the spontaneous state, while from there the system can easily fall directly into the new high activity state. The mirror image of this pattern is observed when switching back from rule 2 to rule 1 (red).

### Quantification of the Attractor Stability of a Wong-Wang-Type Working Memory Model

The transition probability from the rule 1 to the rule 2 attractor state can be formulated using the path-integral *P*(Rule1→Rule2,0,*t*) = ∫*Dx*[exp(-*S*(*x*))] over all possible paths *x*, that connect the rule 1 with the rule 2 attractor state and take a given time *t*. The term *S*(*x*) in the exponential depends on the parameters of the dynamical system, including physiological parameters such as the GABA and NMDA conductances and the noise amplitude as discussed below. It also depends on the path *x* and time *t* and is called the action associated with path *x* [46]. The exponential weighting leads to the fact that the transition probability is dominated by the path with the minimal associated action *S*(*x*_min_). This allows us, given a set of parameters describing the rule module, to calculate the most probable transition path, as shown in Fig 7A and 7B, and the associated minimal action, using a discretization and minimization scheme implemented in MATLAB (cf. Methods; full codes at https://sourceforge.net/projects/mcmc-mp/). The minimal path-integral action in turn gives us a quantitative measure of the stability of the rule attractor states with higher minimal path actions associated with less probable transitions and therefore with more stable attractor states.

### Shaping of the Attractor Landscape by Physiological Parameters

The parameters of the rule module that are actually mediating the changes in attractor depth in our model correspond to the scaling parameters *s*_*NMDA*_ and *s*_*GABA*_ of NMDA and GABA channel conductances and the amplitude *σ*_*rule*_ of noisy fluctuations in the network. The relationship between these physiologically relevant parameters and the resulting dynamical landscape is shown in Fig 8A–8D. An increase in the slow, excitatory NMDA channels stabilizes states of high activity by recurrent connections within the selective (i.e., rule-representing) populations and smoothes out noisy fluctuations, leading to deeper basins of attraction for the states corresponding to the active representation of a task rule in working memory. The inhibitory GABA channels in contrast lead to an increase in global inhibition, thereby destabilizing high activity states and generating a more stable spontaneous state. The noise parameter effectively acts by smoothing out the potential landscape, thereby facilitating transitions between all attractor states. This also manifests in corresponding changes in the minimal actions associated with transitions between the individual states, as shown in Fig 8E–8G.

### Correlation of Minimal Path Integral Action with Error Rates and Reaction Times

By reconstructing the individual potential landscapes of the rule module and minimizing the path-integral action from one activated rule basin to the other (Fig 7), we were able to calculate the individual attractor depth of the rule maintenance module without external inputs for each subject. Table 2 lists correlations of the attractor depth as derived from the fitted model for different task conditions, i.e., baseline, distractor inhibition, and task switch, with behavioral measures. Although there were no significant correlations after correcting for multiple comparisons, there was a strong trend towards larger reaction time costs during switching for persons with deeper attractor basins, which is in line with our theoretical expectations. Also, even though these results are far from significant, it is an interesting observation that across all conditions, there were generally fewer errors in persons with deeper rule attractors.

**Table 2:** Correlations of the individual attractor depth with behavior.

Condition	Mean Reaction Time (Baseline) / Reaction Time Costs (Other)		Error Rate	
	r	p	r	p
Baseline	-0.03	0.89	-0.08	0.74
Distractor	0.25	0.29	-0.18	0.45
Switch	0.47	0.04	-0.22	0.34

### Correlation of Minimal Path Integral Action with Differential Recruitment of a Thalamocorticostriatal Dopamine Network

**Fig 9:**
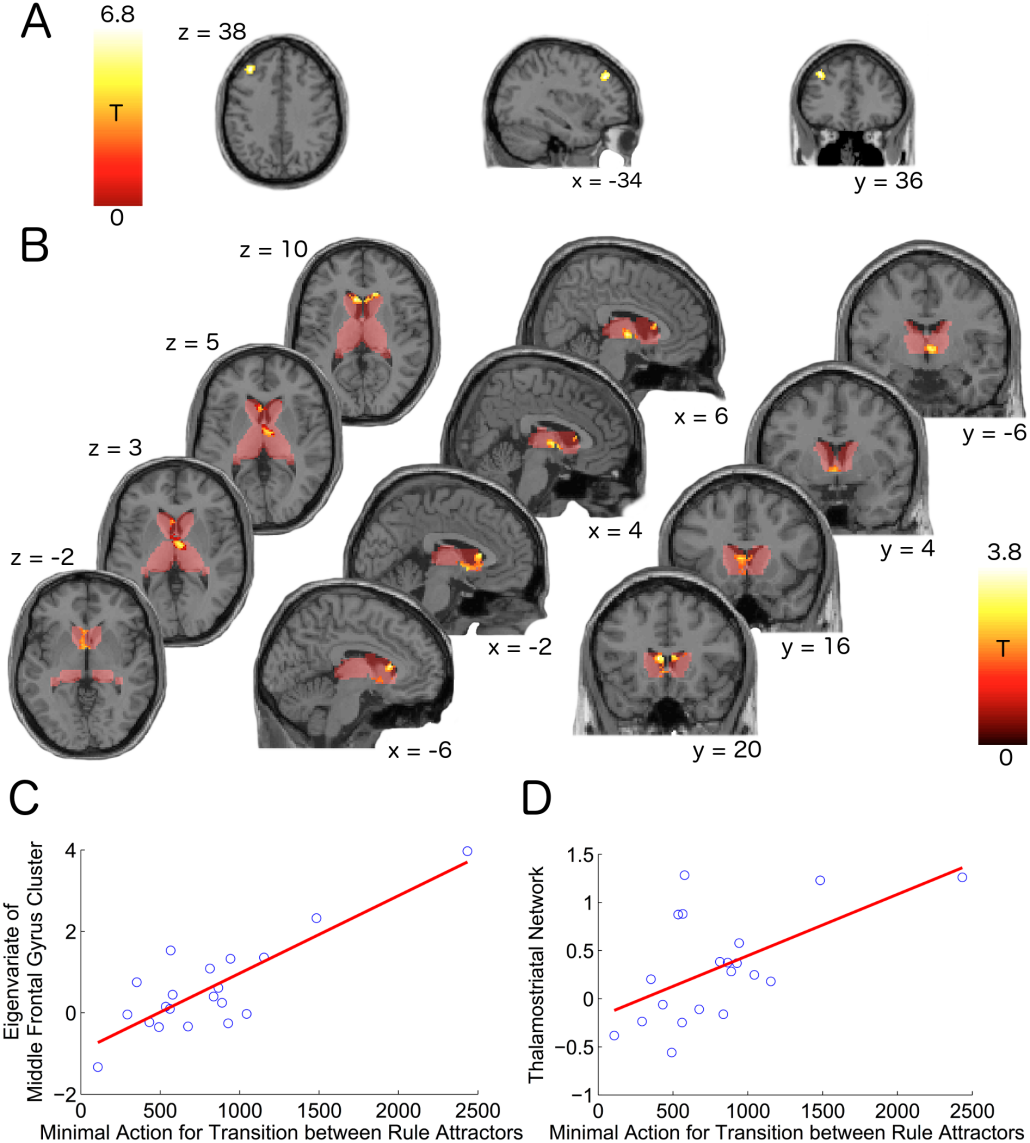
Correlation of the differential recruitment of brain regions in the switch vs. distractor contrast with the individual attractor stability of high activity rule states. (A) Middle frontal gyrus. p = 0.05 cluster threshold, corrected for whole-brain multiple comparisons. (B) Thalamostriatal network. p = 0.05 cluster threshold, corrected for multiple comparisons within an anatomical mask comprising bilateral thalamus and caudate nucleus (red overlay). (C) Eigenvariate of the middle frontal gyrus cluster in the flexibility versus stability condition versus individual minimum action associated with a transition between the rule attractors. (D) Eigenvariate of the thalamostriatal network in the flexibility versus stability condition versus individual minimum action associated with a transition between the rule attractors.

We entered the individual depth of the rule attractors, in terms of the minimal path-integral action for a rule 1 to rule 2 transition, as a second-level covariate for the task switching minus distractor inhibition fMRI contrast. During a task switch maximal flexibility is required, i.e. it makes sense to destabilize working memory attractors to facilitate the switch from one basin to another, and the system has to be externally forced to a new state. In contrast, distractor inhibition requires maximal stability of the networks. So the contrast of these conditions yields regions whose activity has antagonistic effects on cognitive stability and flexibility. Using a cluster level threshold of p < 0.05 (family-wise error correction) as determined with Monte Carlo simulations (AFNI AlphaSim, [48]; using 10.000 iterations), we found a single region in the left middle frontal gyrus (Brodmann area 9), showing higher recruitment in the switch versus distractor condition in subjects with deeper attractor basins (Fig 9A and Table 3).

**Table 3:** Brain regions whose activation in the switch versus distractor contrast is modulated by the individual, i.e., subject-specific estimate of attractor stability.

		*MNI coordinates of peak voxel*				
*Brain region*	*BA*	*x*	*y*	*z*	*# voxels* *	*T*_*max*_
caudate nuclei (bilateral) and thalamus (left)	-	-6	20	10	316	3.79
middle frontal gyrus (left)	9	-34	36	38	86	6.83

Attractor stability is measured by the minimal path integral action for the transition between the rule 1 and rule 2 attractor states.

* voxel size = 2x2x2 mm^3^

The flexible gating of new information into the prefrontal cortex is crucially dependent on thalamocorticostriatal loops [31,32,49–51] and modulatory effects of dopamine in tasks requiring cognitive flexibility are often found in the striatum [15,17,33,52]. Thus, we conducted a second analysis restricted to an anatomical regions-of-interest mask comprising the bilateral thalamus and caudate nuclei (WFU Pickatlas 3.0.4, [53], shown in Fig 9B). Again we used AlphaSim to determine a corrected cluster level threshold of p = 0.05 restricted to this region-of-interest. We found a single thalamostriatal network consisting of a peak in the right thalamus and bilateral centres of activation in the dorsolateral heads of the caudate nuclei, showing the same correlation of higher differential recruitment during task switching versus inhibition for deeper rule attractors (Fig 9B and Table 3). The higher recruitment of this thalamocorticostriatal network is illustrated in Fig 9C and 9D by plotting the eigenvariates that were extracted from the middle frontal gyrus cluster and the thalamostriatal network versus the individual minimal action required for a transition between rule attractors. The eigenvariates correspond to the projection of the subject-specific contrast vector onto the maximum eigenvector of the voxel-covariance matrix across subjects, i.e. the first principal component. This measure takes spatially heterogeneous functional responses within the clusters better into account than a simple mean across the cluster [54].

As is shown in Fig 7A and 7B, the dynamics of the rule module confine the trajectories between the two high-activity rule states to a narrow valley which connects the two states via the basin of attraction of the spontaneous state of the system. This leads to a potential profile along the transition paths corresponding to the minimal action as shown in Fig 7E and 7F. Therefore, the main contributions to the minimal action are the heights of the potential barriers to be crossed during the transition from the active rule state to the basin of attraction of the spontaneous state and between the basin of attraction of the spontaneous state and the new rule state. This explains why there are three model parameters influencing the amount of external forcing needed to switch from one rule to the other: The NMDA conductances (*s*_*NMDA*_) increase the stability of the high-activity rule state, the GABA conductances (*s*_*GABA*_) increase the stability of the spontaneous state, and the diffusion parameter of the Ornstein-Uhlenbeck noise added to the deterministic dynamics of the rule module (*σ*_*rule*_) generally decreases the stability of the rule states and the spontaneous state, as illustrated in Fig 8.

Therefore, those three individual contributions play together in mediating the amount of external forcing needed to switch from one rule state to another. Viewed individually, the correlation of each of these parameters with the eigenvariate of the medial frontal gyrus cluster shown in Fig 9A and 9C is not significant, i.e., *s*_*NMDA*_: r = 0.40, p = 0.08; *s*_*GABA*_: r = 0.29, p = 0.21; *σ*_*rule*_: r = -0.36, p = 0.12. However, all three point towards the direction expected with respect to the corresponding changes in the potential landscape in Fig 8. As these weak correlations suggest, the individual contributions of these three parameters can vary: E.g., the depth of the spontaneous state might be negligible in some subjects (as for example sample subject 1 as shown in Fig 7E) while making a substantial contribution in others (such as sample subject 2; cf. Fig 7F). This leads to a degeneracy of model parameter combinations with respect to the effort required to change the state of the system from one rule attractor to the other. The minimal action, in contrast, integrates all relevant model parameters, and removes this degeneracy, by calculating a theoretically motivated measure of stability. As shown above, it correlates nicely with the activation of a thalamocorticostriatal gating network, that can be seen as a proxy for the external forcing applied to the working memory maintenance system. This, in turn, points to the fact that the relevant dynamical quantity is in fact the stability of the attractor states, which is controlled by—possibly degenerate—combinations of three physiological parameters.

## Discussion

In this work we developed a physiologically plausible model of working memory maintenance and decision making that was able to quantitatively fit individual behavioral data of a task probing cognitive flexibility and cognitive stability. To improve the speed and to facilitate the interpretation of this model, we used a well-known mean field approach to implement the maintenance of the currently active task rule [25]. We furthermore reduced the decision module to a non-linear three-dimensional diffusion process. The latter extends the work of Roxin and Ledberg [26] and thereby shows a canonical way to generalize drift-diffusion models to more than two choices. By combining the analytical reductions with parallel computing on graphics hardware, we developed a Bayesian Markov-Chain-Monte-Carlo fitting framework that is available online (https://sourceforge.net/projects/mcmc-mp/).

The biological nature of our model allowed us to use simulated spike rates to predict the energy consumption and therefore the fMRI BOLD response of the simulated working memory module. Resulting time-courses predicted with high accuracy the activation of a fronto-parietal network, consisting of the left IFJ and the left IPS, which is known to be involved in the cognitive control functions probed here [27–29]. This serves both as intrinsic validation of our model and as a possible explanation of the physiological mechanisms underlying the role of this network in cognitive stability and flexibility. Applying methods from statistical mechanics [46], we were able to visualize the potential landscape of the working memory module and the role of individual physiological parameters. Furthermore, we adapted methods from statistical physics [46,47] and used them to quantify the individual attractor stability of the fitted rule modules, corresponding to the IFJ-IPS network. The individual attractor stability of the working memory network correlated with an increased activation in a thalamocorticostriatal network known to be involved in the flexible gating of new information to prefrontal cortex [31,32] and in the dopaminergic modulation of cognitive stability and flexibility [17,50]. This yields further evidence for the actual role of attractor dynamics underlying neural computations, interindividual differences in cognitive stability and flexibility, and—connecting the physiological parameters of our model with known molecular mechanisms of dopamine action on single cells and the identified macroscopic brain networks—dopaminergic modulation of behavior.

### Drift-Diffusion Models as Approximations of Physiological Noisy Attractor Dynamics

Neurophysiological models of two-choice decision making can be reduced to nonlinear drift-diffusion models [26]. These in turn can be linearized to yield drift-diffusion models as they are often used to fit behavioral data [55]. We show here that the same approach indeed works for four-choice models of decision-making. In this way we can alleviate the arbitrariness in the process of the generalization of drift-diffusion models to many choices, that was pointed out recently [44]. Furthermore, we receive very simple quadratic ordinary differential equations which are fast to evaluate and easy to parallelize and which inherently generate the winner-take-all dynamics of more complex decision networks. Interestingly, the reduced model lives in the minimal dimensional space in which a symmetric embedding of four choices is possible, thereby also enabling an intuitive visualization and understanding of the decision process as shown in Fig 3B and 3E.

Additionally, the neural perspective on drift-diffusion models that is presented here helps to understand why linear drift-diffusion models, such as the Wiener diffusion model [43,56], need additional noise sources beyond the diffusion process itself to really fit individual data well: The Wiener model, for example, describes the continuous accumulation of evidence for the choice between two options as Wiener process (i.e., Brownian motion) with drift rate *ξ* and diffusion coefficient *s* ^2^: *X*_*t*_ = *ξt*+*sW*_*t*_. The process starts at a certain point *z*ϵ(0,*a*) and a decision is made as soon as it crosses the decision boundaries at 0 or *a*. These models are fitted to individual behavioral data via the starting point *z*, the upper decision boundary *a*, and the drift rate *ξ*. However, to quantitatively fit behavioral data well, the drift parameter and starting point can not be constant parameters, instead they are usually fit by a uniform distribution of starting points *z*ϵ[*z*_min_,*z*_max_] and a normal distribution of drift parameters *ξ*ϵ*N*(*v*,*η*) out of which the corresponding parameters are drawn for each simulated trial. While this need to add additional noise sources to the drift-diffusion process beyond the fundamental Brownian motion might seem as a drawback of the formalism, requiring additional arbitrary parameters, the underlying reason becomes immediately obvious as soon as the drift-parameter is understood as an external forcing on a neural network exerted by another part of the brain. More specifically, the decision module in our model receives top-down inputs from the rule module by forwarding the current firing rates *r*_1_,*r*_2_ of the rule representing populations to the corresponding decision selective populations, using the feed-forward weight *c*_+_. This top-down input biases the transition from the spontaneous state to the decisions congruent with the currently active task rule. In our model, the rates *r*_1_,*r*_2_ are simulated using the firing rate equations derived in [25] with additive Ornstein-Uhlenbeck noise. In general, the top-down forcing most probably stems from a region with its own finite size fluctuations and is transmitted by a Poissonian rate code. Therefore, it is completely natural to expect that this forcing is fluctuating and adding an additional noise source to the intrinsic stochastic dynamics of the network. From this perspective it becomes clear that simpler models, which capture such a forcing by a single drift parameter, require a distribution over this parameter to capture the full behavioral variability.

### Attractor Dynamics as Fundamental Building Block of Neural Computations

The concept of stochastic dynamics of neural networks as fundamental building blocks of cognition has been discussed for a long time. Attracting states of neural activity can represent active memories [22,57], the transition from the outer rim of a basin of attraction to its center can explain the auto-associative properties of our memories [58], the transition between different attractor states can model decision-making [59,60], and the passage through more complicated dynamical entities, such as stable heteroclinic channels, has been proposed as a model for the wandering from thought to thought [61,62]. There is also a growing field trying to relate abnormal dynamical properties of brain networks to various psychiatric diseases, such as obsessive-compulsive disorders or schizophrenia [18,19,63,64]. Despite the power and elegance of the stochastic dynamical systems perspective on physiological and pathological brain function [65], there have only been few advances in the direct observation of attractor-like properties of neural ensembles in vivo [20,21]. Most of them relied on high resolution multiple single unit recordings coupled with powerful statistical learning and time series analysis methods. Naturally, the underlying high-resolution recording techniques were highly invasive and can hardly be applied to humans. Complementary to those very data-driven approaches, we tried to model rule based decision making, cue based task switching, as well as distractor inhibition in humans with a minimal, physiologically plausible [22,40] neural network based on stochastic attractor dynamics.

Our model was able to explain the behavior of all subjects in the baseline, switch, and distractor conditions of a complex behavioral paradigm in terms of the decisions taken and the distributions of reaction times. Furthermore, although we fitted this model only to the behavioral data, the biological nature of our model then allowed us to predict the fMRI BOLD time course of the task rule maintenance module, thereby identifying a brain network known to be involved in the cognitive processes examined here, i.e., cognitive flexibility and stability, including the left inferior frontal junction area and the left intraparietal sulcus (see, e.g., [27] for meta-analytic evidence from human functional neuroimaging). The implications of the present work for understanding the computational role of this cortical system for the executive control of behavior will be discussed in the next section. Here, we want to point out that this unexpectedly high level of convergence between the simulated time course of the rule module and the measured time course of the task switching network in our view strongly supports the validity of the present modeling approach.

Finally, a crucial property of the rule attractor system—i.e., the minimal action required for a transition from one rule to the other—was correlated with the specific recruitment of a thalamocorticostriatal network during switching between tasks, i.e., cognitive flexibility. This network was previously shown to be related to flexibility in terms of task switching [32,33,50] and contains striatal regions that critically underlie the flexible change of behavior [5,17,66] and that are modulated by dopamine related genetic polymorphisms and pharmacological interventions [15,52]. Taken together, we think that these results are strong support for the actual computational importance of neural attractor states and transitions in human cognition.

Converging evidence comes from a recent study of top-down guided switching of attention between different object dimensions used to categorize individual stimuli [67]. In that study, subjects had to respond either to the color or the orientation of a colored bar, depending on a task cue which is presented before each stimulus. These authors used a highly similar architecture (see also [34] for the original proposal): The task cue input is applied to an attractor network with two rule-selective populations, which is implemented using the firing rate equations of Wong and Wang [25]. This rule representation network in turn biases the categorization module, which consists of four selective populations tuned to show winner-take-all attractor dynamics. Similar to our work, the top-down bias from the rule network is combined with the bottom up stimulus inputs to the category selective pools to create a forcing towards the high-activity attractor state of the category that is congruent both with the shown stimulus properties and the active task rule. Differentiating this model from the present work, Ardid and Wang [67] added more detailed mechanisms of bottom-up attentional modulation in the form of two ring attractor networks, that implement the perception and representation of the color and orientation properties of the shown stimulus and thus allow for the top-down modulation of attention by biasing the perceptual networks according to the currently active task rule and selected category.

The study by Ardid and Wang [67] differs in further aspects from the present work. For example, their behavioral paradigm did not have a one-on-one mapping of decision to behavioral output, as was the case in our task, which required them to add an additional two-attractor network to project the four possible categorizations to two possible behavioral outputs. Using this model they were able to explain several hallmark features of task-switching behavior observed in humans, such as switch cost, congruency effects, and task-response interactions, and to reproduce single-neuron activity patterns recorded from behaving monkeys solving this task-switching paradigm [67]. In summary, this recent work converges with our results to highlight the top-down bias exerted by a rule-representing attractor network onto both decision making and perceptual systems as fundamental principle underlying the ability to flexibly switch behaviors. Given its slightly different architecture, this work complements our own results. Our study, in turn, differs from the work by Ardid and Wang [67] in that we were able to demonstrate the ability of such a network architecture to fit the full behavior of individual subjects, to demonstrate a potential implementation of such a rule representing working memory network within a specific brain network in humans (i.e., left IFJ and IPS), and to also suggest a neural correlate of a central dynamical property of our network model, i.e., the individual attractor stability, in the activity of a thalamocorticostriatal updating network.

### Computational Role of the IFJ in Task Switching and Distractor Inhibition

There is strong evidence for a crucial role of the IFJ-IPS network in cognitive control. Meta-analyses showed a consistent activation of the left IFJ and IPS in tasks requiring cognitive control, including task-switching and inhibition [27], and a more recent meta-analysis showed domain-general involvement of the IFJ-IPS network in task-switching, independent of the concrete task [28]. These findings were bolstered by a recent fMRI study [29]. Yet, besides specifying the consistent involvement of the network in cognitive control, its exact computational role and the underlying neurocomputational mechanisms have so far not been specified. By constructing a model based on minimal, physiologically plausible assumptions concerning the neural network architecture of the neocortex, we were not only able to recover the full richness of behavior, but also to relate with very high statistical power the computational properties of this network to the macroscopic activation patterns of a frontoparietal network consisting of the left IFJ and regions in the left IPS.

Our work suggests that the IFJ, together with the identified intraparietal regions, forms a fronto-parietal working memory network that represents the currently active task rule by means of stable attractor states of recurrently connected populations of excitatory neurons. More speculative, we suggest that the IFJ encodes the actual task rule while parietal cortex may keep this representation active by means of sustained internal attention [68,69], implemented via stable, high activity attractor states. While our present model-driven fMRI analysis does not dissociate the functional roles of IFJ vs. IPS, this proposal would be in line with recent proposals of emergent working memory mechanisms [68–71], as well as with memory patterns found in studies on visual working memory [72–74]. In those studies, the identified regions include early visual cortex, assumed to represent the actual working memory contents, and posterior parietal regions—consistent with the results of our study—that supposedly keep the perceptual representations in an activated state by means of sustained internal attention implemented by attractor networks. Another possibility would be the segregation of more abstract aspects of the task-rule, which might be represented in the frontal IFJ cluster, from a concrete stimulus-response mapping, which might be stored in the parietal part of the network. This would be consistent with differential effects of causal manipulations of the IFJ and the IPS regions [75]. Both interpretations are consistent with the underlying mechanism of task rule representation by means of different attractor states of the IFJ-IPS network.

### The Dynamical Balance of Cognitive Stability and Flexibility

As discussed above and shown in Fig 8, the NMDA, GABA, and noise parameters shape the attractor landscape of the rule module. While the physiological interpretation of the NMDA and GABA scaling parameters is obvious, the noise can be conceptualized as an increase in fast, excitatory AMPA conductances, leading to a stronger propagation of finite-size fluctuations that are ubiquitously generated within the selective populations themselves, the inhibitory and non-selective population, and within any external population that might project to our simulated network. In biological neurons the activity of NMDA, GABA, and AMPA channels is known to be directly influenced by dopaminergic modulation at the level of single cell recordings [18,76–78]: The activation of D1 type receptors leads to an increase in the activity of NMDA and GABA receptors and decreases AMPAergic transmission, leading to a stabilization of high-activity attractor states and the spontaneous state of prefrontal working memory networks, while the activation of D2/3 type receptors acts antagonistically. This relates dopaminergic modulation in prefrontal cortex explicitly to the stability of the high activity attractor states in our model, with D1 activation leading to more stable rule representing attractor states and D2 activation acting antagonistically. These explicit model predictions can be tested quantitatively in future work, e.g., using pharmacological manipulation, genetic data, or using animal models in a recently established translational version of our paradigm in mice [79].

We were able to quantify the stability of the high-activity attractor states that represent the currently active task rule in the IFJ-IPS working memory network discussed in the previous section using the path-integral approach [80], which was adapted from quantum mechanics to stochastic dynamical systems [46,47]. We correlated the minimal action required to move from one rule attractor to the other with brain activation during cognitive flexibility (i.e., task switching) as compared to cognitive stability (i.e., distractor inhibition), and found a network of regions in the middle frontal gyrus, thalamus, and caudate nuclei that is consistently related to flexible behavior [33]. The lesion of this dorsolateral frontostriatal loop is often accompanied by impairments in tasks requiring cognitive flexibility [32,49,81]. Additionally, modulatory effects of dopamine during the performance of tasks requiring cognitive flexibility are often found in the dorsolateral striatum, i.e., in the caudate head, that we found to be related to the stability of attractor-like task rule representations [15,17,50,52]. This can be interpreted as the need for higher recruitment of a frontostriatal updating or gating [17,30,31] mechanism during task switching versus distractor inhibition in subjects with deeper basins of attraction in the prefrontal-parietal working memory network maintaining the currently active task rule. This is presumably due to the increased stability of frontoparietal working memory representations that require a stronger external forcing to be moved to a different attractor state.

In summary, our model explicitly and quantitatively links established characteristics of changes in the properties of single cells, in terms of AMPA, NMDA, and GABA parameters, to the stability of a frontoparietal working memory network that, as we suggest here, is involved in maintaining the currently relevant task rule in situations requiring cognitive flexibility vs. stability. The stability of these attractor states in turn modulates the activation of a thalamocorticostriatal updating (or gating) loop during cognitive flexibility as compared to cognitive stability. In this way we can connect in a systematic way the findings from electrophysiology, showing dopaminergic modulation of glutamatergic and gabaergic transmission via D1 and D2 type receptors, with high level functional neuroimaging and behavioral results, that have previously shown dopaminergic modulation of both cognitive stability and flexibility [17]. We conclude that part of the dopaminergic modulation of flexible versus stable behavior, as well as individual differences in stability vs. flexibility depends on the fine-grained tuning of the stability of frontoparietal working memory representations of the currently active task rule.

### Conclusions

The results presented above are an example of how an explicit, physiologically constrained model can help to gain insights into the neural dynamics and computations underlying human behavior and brain function. In this way, causes at the molecular level, such as the dopamine action on metabotropic receptors, can be linked to changes in the dynamics of simulated networks via physiological parameters, such as the scaling factors of simulated NMDA and GABA channel conductances or the simulated neural noise. Those dynamics in turn can be used to predict behavior and functional neuroimaging data. By fitting those models explicitly to the data of individual subjects, one can access neurophysiological parameters that are not directly observable in humans, and can find the signatures of minute dynamical changes in data that covers completely different spatial or temporal scales. Here, for example, we were able to ameliorate the crude temporal resolution and the rather indirect observation of neural processes in fMRI by explicitly modeling the time course of a hypothesized task rule module over the full run of the experiment, on the basis of the dynamics of individual neural populations. This allowed us to exploit the formidable coverage and spatial resolution of this neuro-imaging technique to both test our model and constrain the underlying computational role of the IFJ-IPS network. This method of indirect inference by very explicit models is the staple of physical science and, as we show here, now also viable in the neurosciences due to the convergence of developments in cheap parallel computing hardware, scientific computing algorithms, mathematical modeling, behavioral paradigms, neuroimaging techniques, and a continuous increase in the understanding of neural dynamics both at the molecular and cognitive level.

## Methods

### Ethics Statement

The study was approved by the local ethics committee of the medical faculty of the University of Heidelberg (Study ID F095/2011), and all participants gave written informed consent.

### Participants

Twenty-six participants took part in the study. All had normal vision, reported no history of any neurological or psychiatric diseases, and were right-handed. One participant was excluded due to technical problems with response recording. A further five participants were excluded from fMRI data analyses, as their error rates were higher than 30% in at least one condition. Thus, data of 20 participants were entered into the final fMRI analyses (10 men; age = 20–32 years, mean age = 23.5 years).

### Task

The task was established in [12] and is illustrated in Fig 2. Participants had to respond fast and accurately by button presses to digits between 1 and 9 (excluding 5) that were presented in different shades of gray against a black background. Trials had a fixed duration of 2 seconds, with stimuli presented for 900 msec. Responses were registered during the whole trial period.

In 80% of the trials, only one digit was shown above the fixation cross using a constant, medium gray value (127, baseline task). Participants had to decide whether this digit was odd or even and responded with the index/middle finger of the right hand. For the remaining 20% of trials, two digits appeared on the screen, that is, one above and one below the fixation cross. In this case, the gray value (i.e., the relative brightness) of the digits indicated which rule had to be applied: In the distractor inhibition condition, the upper digit was brighter (gray value randomly sampled from the interval [169,195]) than the lower digit (gray value = [255 − gray value of the upper digit]). In this condition, participants were instructed to continue using the odd/even decision rule applied to the upper digit. However, in the task switch condition, the lower digit was brighter (gray value randomly sampled from the interval [169,195]) than the upper digit (gray value = [255 − gray value of the lower digit]), which signaled participants to switch from the upper to the lower digit and to decide whether it was smaller or larger than 5. Participants were instructed and trained such as to always use the brighter of the two digits for task performance. In switch trials, the response had to be given with the index/middle finger of the left hand. After every critical trial (i.e., task switch or distractor inhibition), a series of at least three baseline task trials followed before the next distractor inhibition or task switch trial appeared. Assignment of the task rules to the hands was counterbalanced across subjects.

Finally, in an ambiguous condition, the grayscale values of the two digits were almost identical, such that it was impossible to decide by vision which digit was brighter. Grayscale values of the lower digit were selected randomly from a predefined range (117–137) around the middle grayscale value of the upper digit (which was identical to the one used in the baseline task, i.e., 127). The slight variation of the grayscale values of the ambiguous lower stimuli along a continuum of 8.2% of the brightness range aimed at avoiding that participants consciously categorized this condition as ambiguous, as this could have resulted in strategic response behavior. The reasoning behind the ambiguous condition was to assess the individual participant’s number of task switches in this condition, termed spontaneous switching rate (SSR).

Digits and conditions were presented in a pseudorandomized manner with a minimum of three and a maximum of six baseline task trials presented between two task switch, distractor inhibition, or ambiguous trials.

Before entering the MRI scanner, subjects underwent an instruction and training session lasting approximately 25 min. In this session, the individual tasks were first trained up to a performance criterion (90% correct) independent from each other. Then the complete task, except for the ambiguous condition, was trained until a performance criterion (80% correct) was reached for each condition (baseline, switch, distractor) individually. The ambiguous condition was encountered the first time during task performance in the scanner.

Although the ambiguous condition is conceptually very interesting, it differs in several, crucial aspects from the other conditions of this task, which make it hard to be modelled explicitly: It was not explicitly instructed, not even disclosed to the subjects. Instead, the subjects encountered this novel condition for the first time during task performance. Additionally, it is the only trial type in which there is not enough information to objectively identify a single, definite correct answer. This makes this condition intrinsically more stochastic and also more variable than the well defined, stereotypic baseline, switch, and distractor trials, which manifests in the fact that the variability in reaction times and error rates was found to be highest within this condition [12]. Furthermore, since the ambiguous condition was not trained up to a performance criterion, which was done for all other conditions, there might be substantially more learning involved in this condition. This fundamentally violates the assumption of static synapses that we make in our model. The significant increase in reaction times as compared to the other conditions [12] hints also at the involvement of additional higher order cognitive processes. Taken together, these factors make the ambiguous condition intrinsically more complex and qualitatively different from the baseline, switch, and distractor conditions. While the latter three conditions can be conceptualized in terms of a static, already established and trained working memory and decision making network, without needs to treat learning or higher order action selection processes into account, this does not hold true for the ambiguous condition. For these reasons, we did not include the ambiguous condition in our model.

### Network Architecture

The network architecture used in our simulations is shown in Fig 3A. We implemented ongoing baseline trials, task switching and distractor inhibition by a network consisting of two functional modules, i.e., a rule module and a decision module [34,82]. The *rule module* is a working memory module that is able to actively represent the currently relevant task rule by winner take all dynamics between two selective pools (R1, R2) of excitatory pyramidal cells with strong recurrent synapses, which are embedded into a large pool of shared inhibitory interneurons (I) and non-selective pyramidal cells (NS). This network corresponds to a well-studied working memory network [22]. The *decision module* represents the four relevant decisions (resulting from 2 tasks x 2 decision option, i.e., odd, even, >5, <5) by four pools of excitatory pyramidal cells, which again form a winner take all system by means of strong reverberating synapses and shared inhibition. These networks have been shown to reproduce correct decisions, errors and their corresponding reaction time distributions successfully in two-choice [23,24,83] and multiple-choice [40] decision making.

Both network modules share the same basic architecture of selective pools of recurrently coupled excitatory cells embedded into a shared pool of non-selective excitatory neurons and interneurons. The relative numbers of selective versus non-selective excitatory cells (10%-15%) is consistent with the coding levels of working memory and decision selective neurons found in electrophysiological experiments [59,60,84,85] and the relative amount of inhibitory interneurons (20%). The locally recurrent excitatory architecture corresponds to basic anatomical properties of the cortex [35,36], often associated with canonical cortical microcircuits [37,38].

By the combination of nonlinear, positive feedback within a selective population and global inhibition, these systems can reach several stable states, which fall into two categories: A *single spontaneous state*, in which all selective neurons fire with the same, low firing rate of approximately 3 Hz. This state represents a blank working memory network or a decision network before a decision has been made. In the *high activity states*, one of the selective populations shows high activity that is stabilized by the recurrent excitatory connections and the global inhibition. This high activity leads to a rise in the activity of the inhibitory population, which in turn suppresses the activity of the other populations. This winner-take-all dynamics allows the working memory network to represent a single active rule in working memory and the decision network to reach an unambiguous decision.

Decision making is implemented in this model by the transition of the decision module from its spontaneous state to one of the four decision states corresponding to the dominance of the corresponding selective population. The transition is biased by top down input from the rule module due to feed-forward excitatory synapses from the rule 1 (parity) selective pool to the corresponding decisions (i.e., odd, even) or from the rule 2 (magnitude) selective pool to the corresponding decisions (i.e., >5, <5). This input interacts with bottom up inputs conveying the relevant properties of the presented stimuli, i.e. the parity of the upper and—during the distractor and switch conditions—the magnitude of the lower stimulus. The combined forcings of the top-down rule and bottom-up stimulus inputs drive the decision module towards the correct decision. Still, due to noise in both modules, the system is able to produce all kinds of error types. The exact mechanism of the simulation of individual trials and the generation of behavioral data will be discussed after the reduced dynamics of both modules are described in the following sections.

Our model describes a relatively short experiment (10 minutes) following an extensive training session (>25 minutes, cf. above section Task). Thus the behavioral situation simulated is a state in which participants have already acquired all relevant rules. This allows us to assume that all the learning associated with the creation of rule representations has taken place. Consequently, we neglect learning effects over the course of the experiment, i.e. we work with fixed, static synaptic weights. Furthermore, the recurrent structure of our network is consistent with the assumption of previous formation of associated neural ensembles by Hebbian learning [24].

### Dynamics of the Rule Module

To efficiently simulate the rule module, we use the model of Wong and Wang [25] which is nicely outlined in [39]. These authors started with a neural network consisting of two selective pools of excitatory pyramidal cells (corresponding to the R1, R2 population in our model) embedded into a pool of shared inhibitory interneurons and non-selective pyramidal cells, which consisted of leaky-integrate and fire neurons implementing AMPA, GABA, and NMDA channels, and showed that the dynamics of the mean firing rates (*r*_1_,*r*_2_) and synaptic gating variables (*S*_1_,*S*_2_) of the two selective populations can be described by the two-dimensional dynamical system

$$\begin{array}{l} \frac{dS_{1}}{dt}=-\frac{S_{1}}{\tau_{NMDA}}+(1-S_{1})\gamma r_{1}\\ \frac{dS_{2}}{dt}=-\frac{S_{2}}{\tau_{NMDA}}+(1-S_{2})\gamma r_{2} \end{array}$$

with *γ* = 0.641 and *τ*_NMDA_ = 100*ms*.

These dynamics yield a two-dimensional phase space with three attracting states: A spontaneous state of symmetrically low synaptic activity in the R1 and R2 populations and two states of high synaptic activity in one population and low synaptic activity in the respective other population. The effective potential landscape of this system for two different, physiologically realistic sets of parameters obtained by fitting the model to behavioral data of two participants of our experiment, is shown in Fig 7A and 7B.

The synaptic gating variables are connected to the population firing rates via

$$\begin{array}{l} r_{1}=H_{J_{A,11},J_{A,12}}(x_{1},x_{2})\\ r_{2}=H_{J_{A,22},J_{A,21}}(x_{2},x_{1}) \end{array}$$ (1)

with the effective transfer function

$$H_{J_{A,ii},J_{A,ij}}(x_{i},x_{j})=\frac{a(J_{A,ii})x_{i}-f_{A}(J_{A,ij},x_{j})-b(J_{A,ii})}{1-\text{exp}\left[-d(J_{A,ii})(a(J_{A,ii})x_{i}-f_{A}(J_{A,ij},x_{j})-b(J_{A,ii}))\right]}$$

the parameters

$$\begin{array}{l} a(J_{A,ii})=239400J_{A,ii}+270\left[{\left(VnC\right)}^{-1}\right]\\ b(J_{A,ii})=97000J_{A,ii}+108\left[Hz\right]\\ d(J_{A,ii})=-30J_{A,ii}+0.1540\left[s\right]\\ f_{A}(J_{A,ij},x_{j})=J_{A,ij}(-276x_{j}+106)\theta (x_{j}-0.4)\left[Hz\right], \end{array}$$

and the effective input currents

$$\begin{array}{l} x_{1}=J_{N,11}S_{1}-J_{N,12}S_{2}+I_{0}+I_{1}\\ x_{2}=J_{N,22}S_{2}-J_{N,21}S_{1}+I_{0}+I_{2}. \end{array}$$ (2)

The connections to the physiological parameters of the model is made by the effective connection parameters

$$\begin{array}{l} J_{N,11}=g_{GABA}^{E}\left(\langle V_{E}\rangle -V_{I}\right)\frac{\tau_{GABA}}{1000}C_{I}\frac{c_{I}}{\eta g_{I2}}g_{NMDA}^{eff,I}\langle V_{I}\rangle fC_{E}-g_{NMDA}^{eff,E}\langle V_{E}\rangle fC_{E}w_{+}\\ J_{N,22}=J_{N,11}\\ J_{N,12}=g_{NMDA}^{eff,E}\langle V_{E}\rangle fC_{E}w_{-}-g_{GABA}^{E}\left(\langle V_{E}\rangle -V_{I}\right)\frac{\tau_{GABA}}{1000}C_{I}\frac{c_{I}}{\eta g_{I2}}g_{NMDA}^{eff,I}\langle V_{I}\rangle fC_{E}\\ J_{N,21}=J_{N,12}\\ J_{A,11}=g_{GABA}^{E}\left(\langle V_{E}\rangle -V_{I}\right)\frac{\tau_{GABA}}{1000}C_{I}\frac{c_{I}}{\eta g_{I2}}g_{AMPA}^{I}\langle V_{I}\rangle \frac{\tau_{AMPA}}{1000}fC_{E}-g_{AMPA}^{E}\langle V_{E}\rangle \frac{\tau_{AMPA}}{1000}fC_{E}w_{+}\\ J_{A,22}=J_{A,11}\\ J_{A,12}=g_{AMPA}^{E}\langle V_{E}\rangle \frac{\tau_{AMPA}}{1000}fC_{E}w_{-}-g_{GABA}^{E}\left(\langle V_{E}\rangle -V_{I}\right)\frac{\tau_{GABA}}{1000}C_{I}\frac{c_{I}}{\eta g_{I2}}g_{AMPA}^{I}\langle V_{I}\rangle \frac{\tau_{AMPA}}{1000}fC_{E}\\ J_{A,21}=J_{A,12} \end{array}$$

and the baseline input current

$$\begin{array}{l} I_{0}=lr_{ext}+mr_{ns}+n\psi_{ns}+g_{GABA}^{E}\left(\langle V_{E}\rangle -V_{I}\right)\frac{\tau_{GABA}}{1000}C_{I}\left(\frac{I_{I}}{\eta g_{I2}}-\frac{r_{0}}{\eta }\right)\\ l=g_{GABA}^{E}\left(\langle V_{E}\rangle -V_{I}\right)\frac{\tau_{GABA}}{1000}C_{I}\frac{c_{I}}{\eta g_{I2}}g_{AMPA,ext}^{I}\langle V_{I}\rangle \frac{\tau_{AMPA}}{1000}C_{ext}-g_{AMPA,ext}^{E}\langle V_{E}\rangle \frac{\tau_{AMPA}}{1000}C_{ext}\\ m=g_{GABA}^{E}\left(\langle V_{E}\rangle -V_{I}\right)\frac{\tau_{GABA}}{1000}C_{I}\frac{c_{I}}{\eta g_{I2}}g_{AMPA}^{I}\langle V_{I}\rangle \frac{\tau_{AMPA}}{1000}\left(1-2f\right)C_{E}-g_{AMPA}^{E}\langle V_{E}\rangle \frac{\tau_{AMPA}}{1000}\left(1-2f\right)C_{E}w_{-}\\ n=g_{GABA}^{E}\left(\langle V_{E}\rangle -V_{I}\right)\frac{\tau_{GABA}}{1000}C_{I}\frac{c_{I}}{\eta g_{I2}}g_{NMDA}^{eff,I}\langle V_{I}\rangle \left(1-2f\right)C_{E}-g_{NMDA}^{eff,E}\langle V_{E}\rangle \left(1-2f\right)C_{E}w_{-} \end{array}$$

where

$$\begin{array}{l} g_{NMDA}^{eff,E/I}=\frac{g_{NMDA}^{E/I}}{1+\text{exp}\left(0.062\langle V_{E/I}\rangle \right)/3.57}\\ \psi_{ns}=\frac{\gamma \tau_{NMDA}r_{ns}/1000}{1+\gamma \tau_{NMDA}r_{ns}/1000} \end{array}$$

Here *C*_*E*_ = 0.8, *C*_*I*_ = 0.2 are the relative numbers of excitatory and inhibitory neurons, *C*_*ext*_ = 800 is the number of external, non-selective connections per neuron and *f* = 0.15 is the relative size of the selective populations (the coding level). Further parameters are:

$$\begin{array}{l} c_{E}=310Hz/nA,g_{E}=0.16s,I_{E}=125Hz,c_{I}=615Hz/nA,I_{I}=177Hz,\langle V_{E}\rangle =-53.4mV,\langle V_{I}\rangle =-51.1mV,\\ V_{I}=-70mV,r_{ext}=3Hz,r_{ns}=2Hz,\tau_{GABA}=10ms,g_{I2}=1.7876,r_{0}=11.3721Hz,g_{AMPA}^{ext,E}=0.0021\mu S,\\ g_{AMPA}^{E}=0.1\mu S,g_{NMDA}^{E}=s_{NMDA}\cdot 0.3\mu S,g_{GABA}^{E}=s_{GABA}\cdot 1.3\mu S,g_{AMPA}^{ext,I}=0.00162\mu S,g_{AMPA}^{I}=0.086\mu S,g_{NMDA}^{I}=0.258\mu S,\\ g_{GABA}^{I}=1\mu S,w_{+}=1.68,w_{-}=1-\frac{f(w_{+}-1)}{1-f}=0.88 \end{array}$$

Since the mean-field approach removes the finite-size noise effects, noise is reintroduced using an Ornstein-Uhlenbeck process [25]:

$$\tau_{AMPA}\frac{dS_{noise,i}(t)}{dt}=-S_{noise,i}(t)+\xi (t)\sqrt{\tau_{AMPA}\sigma_{rule}^{2}}$$ (3)

with *τ*_*AMPA*_ = 2*ms*, *ξ*(*t*) Gaussian white noise with zero mean and unit variance, and *σ*_*rule*_ the diffusion parameter of the Ornstein-Uhlenbeck process.

In contrast to the work of Wong and Wang, who added the noise to the synaptic currents and passed it through the f-I-curve (Equations 18 and 19 in [25]), we add the noise directly to the dynamics of the synaptic gating variables $\vec{S}$. This allows us to use the subject-specific fitted parameters to reconstruct and quantify the effective potential landscape of the rule module, as described in the sections "Reconstruction of the Individual Attractor Landscape of the Rule Module" and "Estimation of the Minimal Action for Transitions between Rules" below. This yields the final evolution equations:

$$\begin{array}{l} \frac{dS_{1}}{dt}=-\frac{S_{1}}{\tau_{NMDA}}+(1-S_{1})\gamma r_{1}+S_{noise,1}\\ \frac{dS_{2}}{dt}=-\frac{S_{2}}{\tau_{NMDA}}+(1-S_{2})\gamma r_{2}+S_{noise,2} \end{array}$$ (4)

### Dynamics of the Decision Module

We follow the work of Roxin and Ledberg [26], who showed that neurophysiological models of two-choice decision making [23–25] can be reduced to a one-dimensional nonlinear drift-diffusion process. By using the same approach, we reduce a generic rate model of the mean firing rate of four competing excitatory (*r*_1_,…,*r*_4_) and one shared inhibitory (*r*_*I*_) neural population to a three-dimensional drift-diffusion process. We start with the following set of coupled dynamical equations for the evolution of the mean firing rates *r*_1_,*r*_2_,*r*_3_,*r*_4_,*r*_I_:

$$\begin{array}{l} {\dot{r}}_{1}=-r_{1}+\Phi (sr_{1}-cr_{I}+I+I_{1})+\sigma_{D}\xi_{1}(t)\\ {\dot{r}}_{2}=-r_{2}+\Phi (sr_{2}-cr_{I}+I+I_{2})+\sigma_{D}\xi_{2}(t)\\ {\dot{r}}_{3}=-r_{3}+\Phi (sr_{3}-cr_{I}+I+I_{3})+\sigma_{D}\xi_{3}(t)\\ {\dot{r}}_{4}=-r_{4}+\Phi (sr_{4}-cr_{I}+I+I_{4})+\sigma_{D}\xi_{4}(t)\\ {\dot{r}}_{I}=-r_{I}+\Phi_{I}\left(g(r_{1}+r_{2}+r_{3}+r_{4})+I_{I}\right)+\sigma_{I}\xi_{I}(t) \end{array}$$

where Φ,Φ_*I*_ are nonlinear transfer functions, *s* is the strength of the recurrent coupling, *c* is the inhibitory to excitatory coupling, g is the excitatory to inhibitory coupling, *I*,*I*_*I*_ are the baseline inputs from external non-selective excitatory cells, and *ξ*_*i*_ are noise processes, such as zero mean, unit variance Gaussian white noise or Ornstein-Uhlenbeck processes.

We used a multiple scales Ansatz to develop the dynamics at the bifurcation point of the spontaneous state. For this we examine the linear stability equation at the stable spontaneous state (*R*,*R*,*R*,*R*,*R*_I_):

ddt(Δr→)=(−1+Φ's000−cΦ'0−1+Φ's00−cΦ'00−1+Φ's0−cΦ'000−1+Φ's−cΦ'gΦI'gΦI'gΦI'gΦI'−1)⋅Δr→ (5)

The idea is to find a set of parameters so that the eigenvectors corresponding to the "winner-take-all" instability have zero eigenvalues, while all other eigenvectors have negative eigenvalues. Since the dynamics around a stable fix point follow $\Delta {\vec{v}}_{i}(t)=\Delta {\vec{v}}_{i}(0)e^{\lambda_{i}t}$ for a perturbation along an eigenvector ${\vec{v}}_{i}$ with corresponding eigenvalue *λ*_*i*_, this means that along the directions corresponding to winner-take-all dynamics, the system can evolve freely, while modes corresponding to other eigenvectors decay exponentially. To allow for zero eigenvectors the matrix needs to have a non-zero null-space. This is achieved by choosing Φ’*s* = 1. This yields the following basis of the null-space:

$$v_{1}=\frac{1}{\sqrt{2}}\left(\begin{array}{c} 1\\ -1\\ 0\\ 0\\ 0 \end{array}\right),v_{2}=\frac{1}{\sqrt{2}}\left(\begin{array}{c} 0\\ 0\\ 1\\ -1\\ 0 \end{array}\right),v_{3}=\frac{1}{2}\left(\begin{array}{c} 1\\ 1\\ -1\\ -1\\ 0 \end{array}\right)$$

In this space, solutions can evolve freely. Additionally, looking at the projection of the original basis vectors corresponding to the activity of individual populations

$$\begin{array}{l} \text{pr}\left(\begin{array}{c} 1\\ 0\\ 0\\ 0\\ 0 \end{array}\right)=\frac{1}{\sqrt{2}}v_{1}+\frac{1}{2}v_{3},\text{pr}\left(\begin{array}{c} 0\\ 1\\ 0\\ 0\\ 0 \end{array}\right)=-\frac{1}{\sqrt{2}}v_{1}+\frac{1}{2}v_{3},\\ \text{pr}\left(\begin{array}{c} 0\\ 0\\ 1\\ 0\\ 0 \end{array}\right)=\frac{1}{\sqrt{2}}v_{2}-\frac{1}{2}v_{3},\text{pr}\left(\begin{array}{c} 0\\ 0\\ 0\\ 1\\ 0 \end{array}\right)=-\frac{1}{\sqrt{2}}v_{2}-\frac{1}{2}v_{3} \end{array}$$ (6)

one notes that the directions corresponding to the increase in the activity of a single population form a symmetric tetrahedron, with the spontaneous state at its center.

Using the Ansatz

$$\vec{r}=\left(\begin{array}{c} R\\ R\\ R\\ R\\ R_{I} \end{array}\right)+\underset{\Delta \vec{r}}{\underset{︸}{\varepsilon \left(X(T)v_{1}+Y(T)v_{2}+Z(T)v_{3}\right)+\varepsilon^{2}\left(\begin{array}{c} r_{12}\\ r_{22}\\ r_{32}\\ r_{42}\\ r_{I2} \end{array}\right)}}$$

with T = *εt*, and *I*_*i*_ = *I*+*ε* ^2^ *δI*_*i*_, the second order Taylor expansion of the dynamic equation yields for *O*(*ε* ^2^):

$$\frac{d}{dt}(\Delta \vec{r})=-\frac{\Phi ''}{2}s^{2}\left(\begin{array}{c} \frac{1}{2}X^{2}+\frac{1}{4}Z^{2}+\frac{1}{\sqrt{2}}XZ\\ \frac{1}{2}X^{2}+\frac{1}{4}Z^{2}-\frac{1}{\sqrt{2}}XZ\\ \frac{1}{2}Y^{2}+\frac{1}{4}Z^{2}-\frac{1}{\sqrt{2}}YZ\\ \frac{1}{2}Y^{2}+\frac{1}{4}Z^{2}+\frac{1}{\sqrt{2}}YZ\\ 0 \end{array}\right)-\Phi '\left(\begin{array}{c} \delta I_{1}\\ \delta I_{2}\\ \delta I_{3}\\ \delta I_{4}\\ 0 \end{array}\right)$$

Equating this to Eq (5) using Φ’*s* = 1 yields:

(0000−cΦ'0000−cΦ'0000−cΦ'0000−cΦ'gΦI'gΦI'gΦI'gΦI'−1)︸L⋅(r12r22r32r42rI2)︸r→2=−Φ''2s2(12X2+14Z2+12XZ12X2+14Z2−12XZ12Y2+14Z2−12YZ12Y2+14Z2+12YZ0)−Φ'(δI1δI2δI3δI40)︸N→

Since $v_{i}^{T},i=1,2,3$ is a basis of the left-null eigenspace of **L**, there can only be a solution if $v_{i}^{T}\vec{N}=0,i=1,2,3$. These solvability conditions lead to the final amplitude equations:

$$\begin{array}{l} \frac{d}{dT}X=\frac{1}{2}\Phi '\left(\delta I_{1}-\delta I_{2}\right)+s^{2}\Phi ''XZ\\ \frac{d}{dT}Y=\frac{1}{2}\Phi '\left(\delta I_{3}-\delta I_{4}\right)-s^{2}\Phi ''YZ\\ \frac{d}{dT}Z=\frac{1}{4}\Phi '\left(\delta I_{1}+\delta I_{2}-\delta I_{3}-\delta I_{4}\right)+\frac{1}{4}s^{2}\Phi ''\left(X^{2}-Y^{2}\right) \end{array}$$

These equations govern the deterministic, nonlinear dynamics of our three-dimensional drift-diffusion model at the bifurcation of the spontaneous state, i.e., right when a decision is taking place while the spontaneous state loses its stability. As shown in Fig 3E and 3F, the dynamics show the required winner-take-all behavior which leads to evolution towards the corners of a tetrahedron, corresponding to the increase of activity in one selective population r_i_ and the simultaneous decline in the other selective populations. The equations are symmetric for the exchange of two arbitrary, selective populations.

To gain the required stochastic diffusion behavior we again reintroduce noise using a physiologically plausible Ornstein-Uhlenbeck processes:

$$\tau_{AMPA}=\frac{dI_{noise,i}(t)}{dt}=-I_{noise,i}(t)+\xi (t)\sqrt{\tau_{AMPA}\sigma_{decision}^{2}}$$

with *τ*_*AMPA*_ = 2*ms*, *ξ*(*t*) Gaussian white noise with zero mean and unit variance, and *σ*_*decision*_ the diffusion parameter of the Ornstein-Uhlenbeck process:

$$\begin{array}{l} \frac{d}{dT}X=\frac{1}{2}\Phi '\left(\delta I_{1}-\delta I_{2}\right)+s^{2}\Phi ''XZ+I_{noise,X}\\ \frac{d}{dT}Y=\frac{1}{2}\Phi '\left(\delta I_{3}-\delta I_{4}\right)-s^{2}\Phi ''YZ+I_{noise,Y}\\ \frac{d}{dT}Z=\frac{1}{4}\Phi '\left(\delta I_{1}+\delta I_{2}-\delta I_{3}-\delta I_{4}\right)+\frac{1}{4}s^{2}\Phi ''\left(X^{2}-Y^{2}\right)+I_{noise,Z} \end{array}$$ (7)

Since the aim of our study is to understand the mechanisms of rule representation and their importance for cognitive stability and flexibility, we simplify Eq (7) by introducing the effective parameters *α* = *τ*_*NMDA*_Φ’ and *β* = *τ*_*NMDA*_ *s* ^2^Φ”, and arrive at the final dynamic equations for the decision module:

$$\begin{array}{l} \tau_{NMDA}\frac{d}{dT}X=\alpha \frac{1}{2}\left(\delta I_{1}-\delta I_{2}\right)+\beta XZ+I_{noise,X}\\ \tau_{NMDA}\frac{d}{dT}Y=\alpha \frac{1}{2}\left(\delta I_{3}-\delta I_{4}\right)-\beta YZ+I_{noise,Y}\\ \tau_{NMDA}\frac{d}{dT}Z=\alpha \frac{1}{4}\left(\delta I_{1}+\delta I_{2}-\delta I_{3}-\delta I_{4}\right)+\beta \frac{1}{4}\left(X^{2}-Y^{2}\right)+I_{noise,Z} \end{array}$$ (8)

### Simulation of Behavioral Data

We simulate individual trials by first initializing the rule module at the high activity state of rule 1 (i.e., the parity rule). This is due to the fact that the baseline condition in our task is the response to a single stimulus applying the parity rule. Therefore we assume that subjects have this rule actively represented in working memory at the start of a trial. We do this by initializing the rule module at (*S*_1_,*S*_2_) = (0.8,0.1) and propagating it without noise or external inputs until it has reached the high activity fixpoint. Then we apply two stimuli, namely *I*_1_ and *I*_2_ in Eq (2), encoding the salience of the upper stimulus (associated with the parity rule) and the lower stimulus (associated with the magnitude rule) and propagate the rule module for 900ms, corresponding to the length of stimulus presentation, with the Ornstein-Uhlenbeck noise added. Then we set the stimulus specific input currents to zero and propagate the system for another 1100ms until the end of the trial.

The decision module always starts at its spontaneous state (*X*,*Y*,*Z*) = (0,0,0). During the stimulus presentation, the module receives following inputs

$$\begin{array}{l} \delta I_{1}=D_{11}+c_{+}r_{1}\\ \delta I_{2}=D_{21}+c_{+}r_{1}\\ \delta I_{3}=D_{12}+c_{+}r_{2}\\ \delta I_{4}=D_{22}+c_{+}r_{2} \end{array}$$

where *δI*_*i*_ are defined in Eq (8), *c*_+_ is the forward connection weight from the rule selective populations in the rule module, whose firing rates are denoted *r*_1_,*r*_2_ as in Eq (1), and the corresponding decisions. *D*_*ij*_ corresponds to the bottom up stimulus input of the correct (*i = 1*) and incorrect (*i = 2*) feature in the parity (*j = 1*) and magnitude (*j = 2*) dimension. We simulate decisions by projecting the three-dimensional diffusion process back onto the vectors corresponding to the increase in activity for each selective population as described in Eq (6) and exemplified in Fig 3E. As soon as one of those projections crosses a threshold of 10Hz, we say that the network took the corresponding decision and use the time of threshold-crossing as reaction time. As discussed in [26], the nonlinear dynamics lead to a divergence to infinity in finite time, as shown in Fig 3F. This means that the exact value of the threshold is not relevant, as long as it is far enough from the spontaneous state. So the insensitivity of this simulation approach to the exact threshold value is another advantage of our approach. The inputs applied during the stimulus interval of each trial as a function of condition are shown in Table 4.

**Table 4:** Inputs encoding condition-dependent stimulus information in the rule module.

Condition	Rule 1 Input	Rule 2 Input
**Baseline**	*I*_1_ = *I*_1,base_	*I*_2_ = 0
**Distractor**	$I_{1}=I_{1,base}+{\bar{I}}_{dist}+0.5\Delta I_{dist}$	$I_{2}=I_{1,base}+{\bar{I}}_{dist}-0.5\Delta I_{dist}$
**Switch**	$I_{1}=I_{1,base}+{\bar{I}}_{switch}-0.5\Delta I_{switch}$	$I_{2}=I_{1,base}+{\bar{I}}_{switch}+0.5\Delta I_{switch}$

### Fitted Parameter Set

To encode the three task conditions and the intra-individual behavioral differences, we chose the set of free parameters shown in Table 5. Other parameters were fixed to values shown in Table 6.

**Table 5:** Fitted parameter set.

Parameter	Meaning	prior mean *μ*_*p*_	prior std *σ*_*p*_
*s*_*NMDA*_	Scaling of the g*_NMDA_* parameter.	1.0	0.07
*s*_*GABA*_	Scaling of the g*_GABA_* parameter.	1.0	0.07
*I*_1,base_	Input to rule 1 selective population during stimulus presentation in the baseline condition	0.0	0.07
*D*_11_	Bottom up drift towards the correct parity choice due to stimulus presentation.	0.0	0.07
*D*_12_	Bottom up drift towards the correct magnitude choice due to stimulus presentation.	0.0	0.07
${\bar{I}}_{dist}$	Additional input to parity and magnitude selective rule populations during distractor trial.	0.0	0.07
Δ*I*_dist_	Differential input to parity and magnitude selective rule populations during distractor trial.	0.0	0.07
${\bar{I}}_{switch}$	Additional input to parity and magnitude selective rule populations during switching trial.	0.0	0.07
Δ*I*_switch_	Differential input to parity and magnitude selective rule populations during switching trial.	0.0	0.07
*c*_+_	Feedforward weight from rule pools to corresponding decisions.	0.0	0.07
*σ*_rule_	Diffusion parameter of Ornstein-Uhlenbeck processes implementing noise in the rule module.	0.0	0.07
*σ*_decision_	Diffusion parameter of Ornstein-Uhlenbeck processes implementing noise in the decision module.	0.0	0.07
*β*	Relative strength of deterministic dynamic term in dynamics of the decision module.	0.0	0.07

**Table 6:** Fixed parameter values.

Parameter	Meaning	Fixed Value
*D*_21_	Bottom up drift towards the wrong parity choice due to stimulus presentation.	0.0
*D*_22_	Bottom up drift towards the wrong magnitude choice due to stimulus presentation.	0.0
*α*	Scaling parameter of the external inputs to the decision module.	1.0

### Bayesian Posterior Probability for Model Fitting

To quantify the fit of a given parameter set $\vec{\theta }$ of the model, we simulated 1024 trials of each condition (baseline, distractor, switch) with the given parameter set and calculated the relative probabilities for each possible decision *D* as a function of the task condition *C*, $P_{C}^{SIM}(D)$. For each combination of condition and decision, we recovered the cumulative distribution function of reaction times $P_{C,D}^{SIM}(RT<t)=F_{C,D}^{SIM}(t)$. To compare this to the behavioral data $\vec{X}$ of a single subject, we counted the number of each decision alternative taken as a function of condition $n_{C}^{SUBJECT}(D)$ and calculated the corresponding cumulative reaction time distributions $P_{C,D}^{SUBJECT}(RT<t)=F_{C,D}^{SUBJECT}(t)$. With this we can define the likelihood of the data, given the model parameters by:

$$P(\vec{X}|\vec{\theta })=\prod_{C}M(n_{C}^{SUBJECT},P_{C}^{SIM})\times \prod_{\left(C,D\right)}KS_{2}(F_{C,D}^{SIM},F_{C,D}^{SUBJECT})$$

where *M*(*n*,*P*) is a multinomial distribution and *KS*_2_(*F*_1_,*F*_2_) is the two-sample Kolmogorov-Smirnov probability of two discrete samples stemming from the same underlying distribution. We decided for the two-sample Kolmogorov-Smirnov test statistic $D=\underset{t}{\text{sup}}\left|F_{1}(t)-F_{2}(t)\right|\sqrt{\frac{n_{SIM}n_{SUBJECT}}{n_{SIM}+n_{SUBJECT}}}$, where *n*_*SIM*_,*n*_*SUBJECT*_ are the number of points in each sample, since this test does not assume any specific form of the underlying distribution. From this, one can calculate the probability *KS*_2_ using a Brownian Bridge as Null hypothesis:

$$KS_{2}=\frac{\sqrt{2\pi }}{D}\sum_{k=0}^{\infty }\text{exp}\left(\frac{-{\left(2k-1\right)}^{2}\pi^{2}}{8D^{2}}\right)$$

By combining the likelihood with Gaussian shrinkage priors $P(\theta_{i})=\frac{1}{\sqrt{2\pi }\sigma_{\theta_{i}}}\text{exp}\left(-\frac{{\left(\theta_{i}-\mu_{\theta_{i}}\right)}^{2}}{2\sigma_{\theta_{i}}^{2}}\right)$, to prevent overfitting by "penalizing" large parameter values, one can formulate the full Bayesian posterior distribution using Bayes' Theorem:

$$P(\vec{\theta }|\vec{X})=P(\vec{X}|\vec{\theta })\cdot P(\vec{\theta })/P(\vec{X})$$

with the prior distribution over behavioral data $P(\vec{X})$, which is a priori unknown. Since we are going to employ a Markov-Chain-Monte-Carlo sampling approach, the normalization constant $P(\vec{X})$ can be ignored and we just need $P(\vec{\theta }|\vec{X})\propto P(\vec{X}|\vec{\theta })\cdot P(\vec{\theta })$, with

$$P(\vec{X}|\vec{\theta })\cdot P(\vec{\theta })=\prod_{C}M(n_{C}^{SUBJECT},P_{C}^{SIM})\times \prod_{\left(C,D\right)}KS_{2}(F_{C,D}^{SIM},F_{C,D}^{SUBJECT})\times \prod_{i}\frac{1}{\sqrt{2\pi }\sigma_{\theta_{i}}}\text{exp}\left(-\frac{{\left(\theta_{i}-\mu_{\theta_{i}}\right)}^{2}}{2\sigma_{\theta_{i}}^{2}}\right)$$

### Markov-Chain-Monte-Carlo Sampling Scheme and MAP Estimation

To sample from the Bayesian Posterior Distribution, we used the Metropolis-Hastings algorithm [86,87] with a homogeneous, Gaussian proposition distribution

$$P({\vec{\theta }}^{prop}|\vec{\theta })=\prod_{i}\frac{1}{\sqrt{2\pi }\sigma_{MH}}\text{exp}\left(-\frac{{\left(\theta_{i}^{prop}-\theta_{i}\right)}^{2}}{2\sigma_{MH}^{2}}\right)$$

using *σ*_*MH*_ = 0.01.

For each subject we ran two chains with 1,000,000 steps per chain. This was only possible by implementing the single-trial simulations highly parallel on graphics hardware, using custom developed C++ code based on the NVIDIA CUDA API. Running on a consumer GPU (NVIDIA GTX Titan, 2688 CUDA cores) the fitting of a single subject took about three hours. After discarding the first 50,000 steps of each chain to allow for sufficient burn-in, we combined both chains and estimated the maximum a-posteriori (MAP) parameter set using a locally adaptive kernel density estimator (http://www.mathworks.com/matlabcentral/fileexchange/37374-locally-adaptive-kernel-density-estimation, [88]). The resulting posterior distributions and the locally adaptive kernel density estimates are shown in S1 Fig. The full code can be accessed at http://sourceforge.net/projects/mcmc-mp/.

### Quantification of Goodness-of-Fit

For each individual we quantified the goodness of fit of the decision distributions by performing tests on the simulated versus the experimental decision distributions in the baseline, distractor, and switch condition. We used an exact multinomial test for each condition, giving the probability that the empirically measured distribution ${\vec{x}}_{0}$ of decisions or a more extreme (i.e., less probable) distribution was realized, assuming a multinomial distribution with probability parameters *π*_*i*_ given by the simulated decision distributions using the Bayesian MAP parameter estimates. This can be calculated as $P=\sum_{\vec{x}:P(\vec{x})\leq P({\vec{x}}_{0})}P(\vec{x})$ with $P(\vec{x})=N!\prod_{i=1}^{k}\frac{\pi_{i}^{x_{i}}}{x_{i}!}$, the vector of probabilities $\vec{\pi }$ given by the relative number of decisions taken by the fitted model in each condition, and the vector $\vec{x}$ representing a possible experimental decision distribution, i.e. $0\leq x_{i}\leq N,\sum_{i=1}^{k}x_{i}=N$ for *N* trials with *k* possible choices.

We calculated the probabilities for each of the three conditions, using *k* = 4,*N*_*baseline*_ = 240,*N*_*distractor*_ = *N*_*switch*_ = 20.

Additionally, we calculated for each of the realized reaction time distributions the two-sample Kolmogorov-Smirnow probability *KS*_2_(*RT*_sim_,*RT*_exp_) of the simulated and the experimentally obtained reaction time distributions. This gives the probability of the two samples being drawn from the same underlying distribution.

### Reconstruction of the Individual Attractor Landscape of the Rule Module

To visualize the individual stability of attractor representations in the rule module, we reconstructed the potential landscape corresponding to the individual dynamics in the *S*_1_,*S*_2_-space using the approach of [47], as shown in Fig 7A–7D. These authors showed that in non-conservative stochastic systems that do not fulfill the detailed balance equation, such as our rule-module, it is still possible to define a potential *U* = -ln*P*_ss_ in the Boltzmann sense. This can be done by reformulating the dynamics in terms of the evolution of probability densities on phase space instead of single trajectories. In the case of our rule module these equations can be derived starting from Eq (4),

$$\dot{\vec{S}}=\vec{F}(\vec{S})+{\vec{S}}_{noise}$$

with $\vec{F}(\vec{S})=\left(\begin{array}{c} -\frac{S_{1}}{\tau_{NMDA}}+(1-S_{1})\gamma r_{1}\\ -\frac{S_{2}}{\tau_{NMDA}}+(1-S_{2})\gamma r_{2} \end{array}\right)$, and ${\vec{S}}_{noise}=\left(\begin{array}{c} S_{noise,1}\\ S_{noise,2} \end{array}\right)$ Ornstein-Uhlenbeck processes as defined in Eq (3). These dynamics can be seen as a stochastic, overdampened limit of Newton's second law with the deterministic force $\vec{F}$ and noise term ${\vec{S}}_{noise}$.

Using the fact that the steady-state distribution of the Ornstein-Uhlenbeck process Eq (3) is

$$P(S_{noise,i},t\to \infty )=\frac{1}{\sqrt{2\pi }\sigma_{ss}}\text{exp}\left(-\frac{S_{noise,i}^{2}}{2\sigma_{ss}^{2}}\right)$$

with $\sigma_{ss}^{2}=\frac{\sigma_{rule}^{2}}{2}$ we can approximate the dynamic equation close to the steady state (*t*→∞) by

$$\vec{S}=\vec{F}(\vec{S})+\sqrt{2D}\vec{\xi }(t)$$ (9)

with $D=\frac{\sigma_{ss}^{2}}{2}=\frac{\sigma_{rule}^{2}}{4}={\left(\frac{\sigma_{rule}}{2}\right)}^{2}$ and *ξ*_*i*_(*t*) zero mean, unit variance Gaussian noise.

The corresponding Fokker-Planck-Equation is

$$\frac{\partial P(\vec{S},t)}{\partial t}+\vec{\nabla }\cdot \vec{J}(\vec{S},t)=0$$

with the probability density *P* on phase space, and the flux

$$\vec{J}(\vec{S},t)=\vec{F}(\vec{S})\cdot P(\vec{S},t)-D\vec{\nabla }P(\vec{S},t)$$

When the system reaches its steady state *P*_*ss*_ with $\frac{\partial P_{ss}}{\partial t}=0$, the divergence of the flux vanishes, $\vec{\nabla }\cdot {\vec{J}}_{ss}=0$. This means that we can write the force $\vec{F}$ as:

$$\vec{F}=D\frac{\vec{\nabla }P_{ss}}{P_{ss}}+\frac{{\vec{J}}_{ss}}{P_{ss}}=-D\vec{\nabla }(\underset{U}{\underset{︸}{-\text{ln}P_{ss}}})+\frac{{\vec{J}}_{ss}}{P_{ss}}$$

In this way, the force can be decomposed into the curl-free negative gradient of a generalized potential $-D\vec{\nabla }U$ and the curl-field $\frac{{\vec{J}}_{ss}}{P_{ss}}$, where ${\vec{J}}_{ss}=\vec{\nabla }\times \vec{A}$. The so defined generalized potential fulfills the Boltzman relation *U* = -ln*P*_ss_. This allows for the direct visualization of the potential landscape in terms of the steady-state distribution of the system.

To generate individual potential landscapes we integrated the Fokker-Planck Equation numerically, starting with a homogeneous distribution, and using an implicit back-in-time centered-in-space (BTCS) finite differences scheme:

$$\begin{array}{l} \frac{P^{n+1}-P^{n}}{\Delta t}=-\left(\vec{\nabla }\vec{F}\right)\cdot P^{n+1}-\vec{F}\cdot \left(\vec{\nabla }P^{n+1}\right)+D\Delta P^{n+1}\\ \Rightarrow \frac{P^{n+1}-P^{n}}{\Delta t}=-\left(\left(\vec{\nabla }\vec{F}\right)-\vec{F}\cdot \vec{\nabla }+D\Delta \right)P^{n+1}\\ \Rightarrow \left(\Delta t\left(\left(\vec{\nabla }\vec{F}\right)-\vec{F}\cdot \vec{\nabla }+D\Delta \right)+1\right)P^{n+1}=P^{n}\\ \Rightarrow P^{n+1}={\left(\Delta t\left(\left(\vec{\nabla }\vec{F}\right)-\vec{F}\cdot \vec{\nabla }+D\Delta \right)+1\right)}^{-1}P^{n} \end{array}$$

with

$$\begin{array}{l} {\left(\vec{\nabla }\vec{F}\right)}_{x,y}=\frac{F_{x+1,y}^{1}-F_{x-1,y}^{1}}{2\Delta x}+\frac{F_{x,y+1}^{2}-F_{x,y-1}^{2}}{2\Delta y}\\ {\left(\vec{F}\vec{\nabla }P^{n+1}\right)}_{x,y}=F_{x,y}^{1}\frac{P_{x+1,y}^{n+1}-P_{x-1,y}^{n+1}}{2\Delta x}+F_{x,y}^{2}\frac{P_{x,y+1}^{n+1}-P_{x,y-1}^{n+1}}{2\Delta y}\\ {\left(D\Delta P^{n+1}\right)}_{x,y}=D\left(\frac{P_{x+1,y}^{n+1}-2P_{x,y}^{n+1}+P_{x-1,y}^{n+1}}{{\left(\Delta x\right)}^{2}}+\frac{P_{x,y+1}^{n+1}-2P_{x,y}^{n+1}+P_{x,y-1}^{n+1}}{{\left(\Delta y\right)}^{2}}\right) \end{array}$$

We combined the BTCS finite differences scheme with a fixed boundary condition (*P* = 0) and used the MATLAB 2013 sparse linear algebra implementation to propagate the probability density until it reached its steady state. Due to the stable convergence of the implicit integration scheme and the therefore possible large step-sizes, the calculation of individual potential landscapes took less then 10 minutes for each participant.

We want to emphasize that the fitted *s*_*NMDA*_,*s*_*GABA*_,*σ*_*rule*_ parameters in fact control the individual shape of the potential landscape, as shown in Fig 8. All of these parameters are physiologically meaningful and their physiological counterparts are known to be influenced by dopaminergic modulation [18]. The full code can be accessed at http://sourceforge.net/projects/mcmc-mp/

### Estimation of the Minimal Action for Transitions between Rules

We used the path-integral formalism [80] as it was applied to stochastic gene-expression networks in cell differentiation [46] to quantify the depth of the potential minima corresponding to the activity of a single rule in the phase space of the rule module. The paths corresponding to the most likely, i.e. minimal action, transition between the two high-activity rule states are shown in Fig 7A and 7B. The underlying potential landscapes were calculated from the fitted *s*_*NMDA*_,*s*_*GABA*_,*σ*_*rule*_ parameters.

Starting from Eq (9), the probability of starting at a point ${\vec{S}}_{0}$ at *t* = 0 and arriving at ${\vec{S}}_{1}$ at time *t* can be written as

$$P({\vec{S}}_{1},t,{\vec{S}}_{0},0)=\int Dx\text{exp}(-S(x))$$

The integral is over all possible paths **x** connecting the two states and the contribution of an individual path is given by its action **S**(**x**). Due to the exponential weighting of the individual paths by their negative action, the transition is dominated by the path associated with the minimum action. Practically, the dominant path is calculated by minimizing the action in the Hamilton-Jacobi framework. This eliminates the explicit dependency of the action on time and makes fast optimization possible without problems due to the passage of the system through meta-stable states [46]. The action in the Hamilton-Jacobi framework can be written as

$$S(x)=\int_{{\vec{S}}_{0}}^{{\vec{S}}_{1}}\left(\frac{1}{D}\sqrt{E_{eff}+V(\vec{S})}-\frac{1}{2D}F_{l}\right)dl.$$

Here *dl* is the length of the line element $d\vec{l}$, $F_{l}=\vec{F}d\vec{l}/dl$, $V(\vec{S})=\frac{1}{4D}{\left|\vec{F}(\vec{S})\right|}^{2}+\frac{1}{2}\vec{\nabla }\vec{F}(\vec{S})$, and *E*_*eff*_ is a free parameter determining the total transition time via

$$t_{1}-t_{0}=\int_{{\vec{S}}_{0}}^{{\vec{S}}_{1}}dl\sqrt{\frac{1}{4D(E_{eff}+V(\vec{S}))}}$$

Following [46], we chose $E_{eff}=-V({\vec{S}}_{\text{min}})$ as the minimum of $V(\vec{S})$, corresponding to the longest kinetic time. By choosing ${\vec{S}}_{0},{\vec{S}}_{1}$ as the minima of the potential *U* = -ln*P*_ss_ (cf. previous section) corresponding to the high activity states of the parity and magnitude rule, we calculated the minimal action by minimizing a discretized version of the action using custom Matlab scripts and Matlab's fminunc routine, again closely following [46]. We used a discretization of 50 line elements with an additional penalty of 10^5^ on the variance in the length of individual line elements. The resulting minimal action of the dominant path quantifies the stability of the high activity states of the rule module, since it represents the action corresponding to the easiest and therefore most probable spontaneous transition between the rule 1 (parity) and rule 2 (magnitude) rule attractor states. The fitted parameters moderating the individual shape of the potential landscapes and therefore also the individual minimal actions are *s*_*NMDA*_,*s*_*GABA*_,*σ*_*rule*_ which can all be physiologically interpreted and are shown to be influenced by dopaminergic modulation [18]. The full code can be accessed at http://sourceforge.net/projects/mcmc-mp/

### fMRI Acquisition and Preprocessing

All images were acquired on a 3-T Siemens Trio MRI scanner equipped with a fast gradient system for EPI and a 32-channel head coil. Participants were instructed to lie as quiescently as possible, and their heads were additionally stabilized with cushions. Three hundred ten functional volumes were acquired in a single run lasting approximately 10.5 min, using a T2*-weighted BOLD-sensitive gradient-echo, EPI sequence with 32 oblique axial slices (thickness = 3 mm, interslice gap = 1 mm, field of view = 192 mm, matrix size = 64 × 64, in-plane resolution = 3 × 3 mm, repetition time = 2000 msec, echo time = 30 msec, flip angle = 80°). The first three volumes were discarded to allow for stable magnetization. In addition, a T1-weighted magnetization prepared-rapid gradient echo scan (MPRAGE) was acquired (thickness = 1 mm, field of view = 256 mm, matrix size = 256 × 256, in-plane resolution = 1 × 1 mm, repetition time = 1570 msec, echo time = 2.63 msec, flip angle = 30°).

fMRI data were analyzed using the Statistical Parametric Mapping software (SPM8, http://www.fil.ion.ucl.ac.uk/spm/software/spm8/). EPI images were first slice-time and motion corrected. Segmentation of the structural image provided normalization parameters that were used to normalize the functional images to the Montreal National Institute (MNI) template reference brain. Finally, images were smoothed with an 8-mm FWHM Gaussian kernel.

### Generation and Localization of fMRI Timeseries from Fitted Models

The process is illustrated in Fig 5. For each subject, the energy consumption in the rule module as a function of condition and decision taken was estimated by simulating 1024 trials of each condition using the maximum a posteriori estimate of the optimal parameter set (see above section MCMC Sampling Scheme and MAP Estimation). The individual trials were sorted by condition and decision taken and the average integral of the total spikes in the selective populations over the course of each trial was calculated. The resulting total spike counts were averaged over all trials corresponding to a specific combination of condition and decision. Then the average total spike counts were normalized to the average total spike counts during a correct baseline trial (Fig 5A). Then these estimates were combined with the behavioral log files to construct estimates of the fMRI time courses: First, a hypothetical time course of energy consumption was assembled. For each trial the normalized total spike count was used to estimate the energy consumption depending on which condition the subject had encountered and which decision it had taken (Fig 5B).

Subsequently, this hypothetical time course was z-transformed and convolved with the canonical hemodynamic response function implemented in SPM8 (Fig 5C), resulting in an individual prediction of the fMRI BOLD signal for each participant (Fig 5D). This time course was entered as a regressor in a voxel-wise general linear model of the hemodynamic response using SPM8. We included additional regressors for each individual ambiguous trial and 6 regressors reflecting the motion parameters derived from the preprocessing (Fig 5E). The ambiguous trials were modeled by individual regressors because this is the most flexible and therefore—in terms of variance explained by the rule module regressor—the most conservative way to include their contribution to the BOLD timeseries. We did not include the ambiguous trials in our explicit neural model, since they possibly involve higher order cognitive processes and learning, which were not part of our model (cf. above section Task). The resulting beta-maps of the first level analyses were entered into a standard SPM8 random effects model group analysis.

We chose a single-voxel threshold of *p* = 10^–7^, corresponding to *p* = 0.05 Bonferroni corrected for the number of voxels, combined with a cluster threshold of *k* = 50 for the random effects model group map. We visualized the resulting SPM t-map using Caret 5.65 (http://www.nitrc.org/projects/caret/, [89]) and xjView 8.12 (http://www.alivelearn.net/xjview) as shown in Fig 6.

### Localization of Functional Correlates of Individual Attractor Stability

To estimate the average activation as a function of task condition, a general linear model using a canonical hemodynamic response function and a high-pass filter with 128 sec cutoff [90] was applied including one regressor for the task switch and one for the distractor inhibition condition. The ambiguous condition was split into two regressors, one modeling those trials in which participants had switched and one in which they had chosen to stay with the baseline task. In addition, one regressor was entered for error trials, and six regressors modeled the motion parameters derived from the preprocessing. The baseline task trials served as implicit baseline [91] and were not modeled explicitly. The models were estimated using SPM8.

For the analysis of individual differences, the individual attractor depth in terms of the minimal action associated with the transition between the two high activity states of the rule module (see above section “Estimation of the Minimal Action for Transitions between Rules” for more details) was entered as covariate into the group-level random-effects model of the task switch minus distractor inhibition contrast and the corresponding T-map was estimated using SPM8.

We applied Monte Carlo simulations (AFNI AlphaSim, [48], http://afni.nimh.nih.gov/afni/doc/manual/AlphaSim) to derive a statistical threshold of p < .05 corrected for family-wise error, either across the whole brain or for anatomically selected regions of interest (as described in the Results section where applicable).

## Supporting Information

S1 Fig Sampled Bayesian posterior distribution of model parameters for subject 9. We used a Markov-Chain-Monte-Carlo algorithm [86,87] to sample the Bayesian posterior probability function of model parameters given the individual data of each participant (displayed here for subject 9). The sampled distribution was marginalized for each parameter and then smoothed using an optimal bandwidth kernel density estimator [88], as shown here. These marginalized distributions yield maximum a posteriori (MAP) estimates of the fitted parameters (red). The full width at half maximum (FWHM) of each marginalized distribution is shown in green.
(PDF)

S2 Fig Comparison of behavioral data with fitted models for subjects 2–13. Behavioral data are shown in black, fitted models in orange; compare Fig 4.
(PDF)

S3 Fig Comparison of behavioral data with fitted models for subjects 15–26. Behavioral data are shown in black, fitted models in orange; compare Fig 4.
(PDF)

S4 Fig Decision distributions and mean reaction times of the whole sample and corresponding model fits. To demonstrate that also the group-averaged behavior (often considered in cognitive and neurocognitive research) is well captured by the current model, we here report also averages of empirical as well as fitted performance data. Behavioral data of n = 20 subjects are shown in black, simulated data generated from fitted models is shown in orange. Error bars represent the standard error of the mean.
(PDF)
