# Supplementary material for: Stochastic Dynamics Underlying Cognitive Stability and Flexibility
Source: PLoS Comput Biol. 2015 Jun 12;11(6):e1004331. doi: 10.1371/journal.pcbi.1004331 (PMC4466596; doi:10.1371/journal.pcbi.1004331)

S1 Fig. Sampled Bayesian posterior distribution of model parameters for subject 9. We used a Markov-Chain-Monte-Carlo algorithm [86,87] to sample the Bayesian posterior probability function of model parameters given the individual data of each participant (displayed here for subject 9). The sampled distribution was marginalized for each parameter and then smoothed using an optimal bandwidth kernel density estimator [88], as shown here. These marginalized distributions yield maximum a posteriori (MAP) estimates of the fitted parameters (red). The full width at half maximum (FWHM) of each marginalized distribution is shown in green.

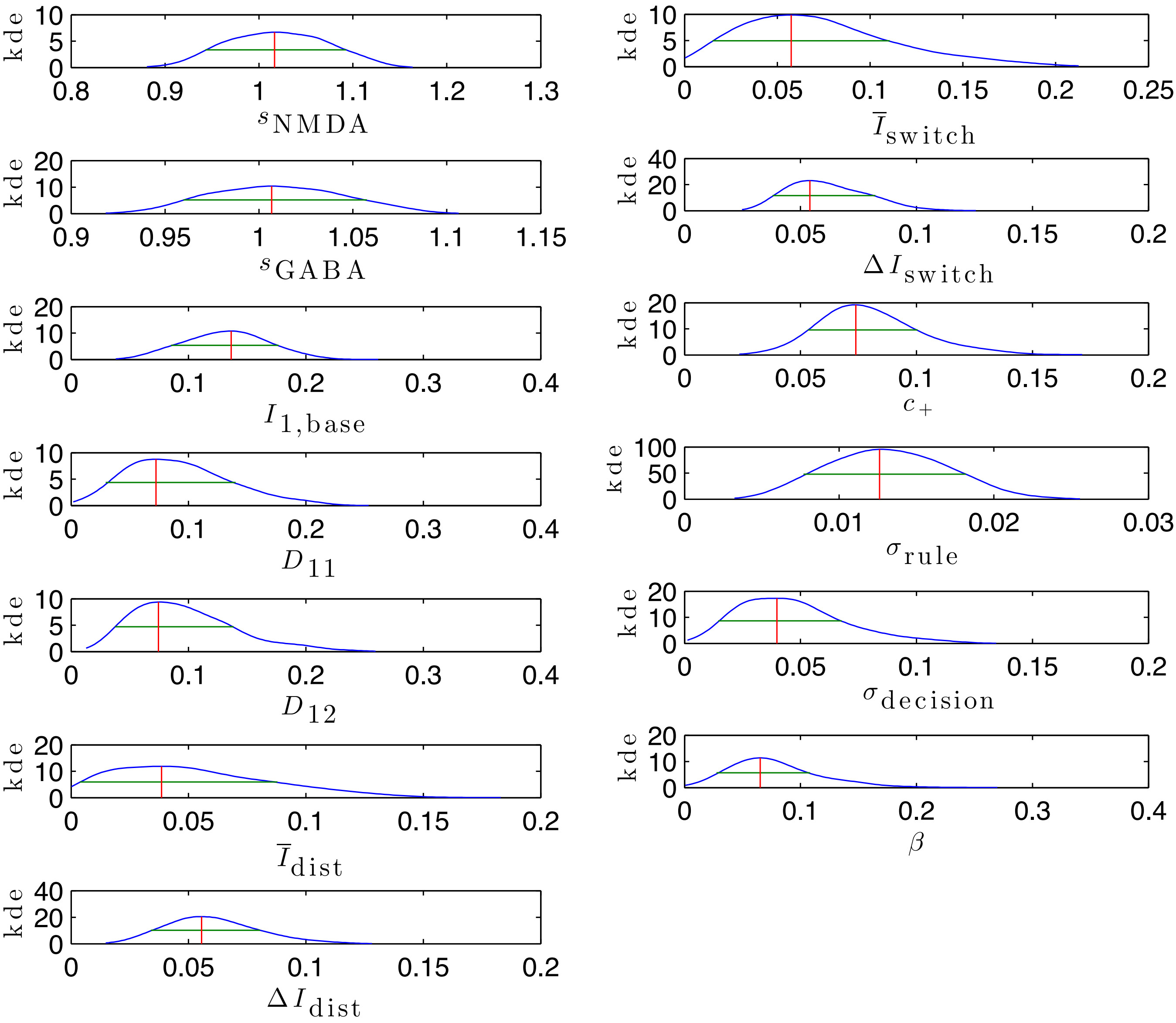

Supplement: S1 Fig — We used a Markov-Chain-Monte-Carlo algorithm [86,87] to sample the Bayesian posterior probability function of model parameters given the individual data of each participant (displayed here for subject 9). The sampled distribution was marginalized for each parameter and then smoothed using an optimal bandwidth kernel density estimator [88], as shown here. These marginalized distributions yield maximum a posteriori (MAP) estimates of the fitted parameters (red). The full width at half maximum (FWHM) of each marginalized distribution is shown in green. (PDF) [file pcbi.1004331.s001.pdf]
