# Supplementary material for: Stochastic Dynamics Underlying Cognitive Stability and Flexibility
Source: PLoS Comput Biol. 2015 Jun 12;11(6):e1004331. doi: 10.1371/journal.pcbi.1004331 (PMC4466596; doi:10.1371/journal.pcbi.1004331)

S3 Fig. Comparison of behavioral data with fitted models for subjects 15-26.

Behavioral data are shown in black, fitted models in orange; compare Figure 4.

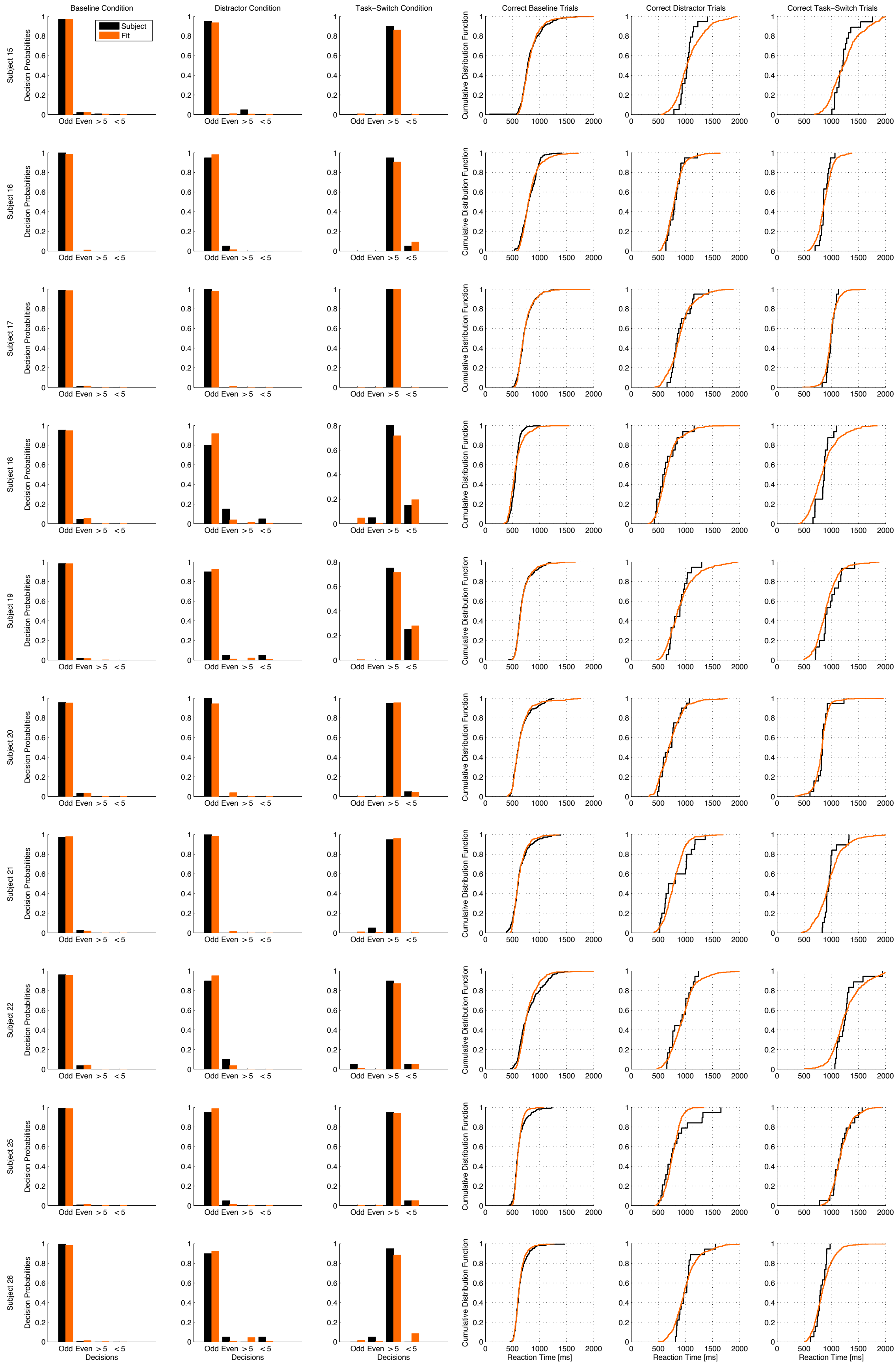

Supplement: S3 Fig — Behavioral data are shown in black, fitted models in orange; compare Fig 4. (PDF) [file pcbi.1004331.s003.pdf]
