# Supplementary material for: Stochastic Dynamics Underlying Cognitive Stability and Flexibility
Source: PLoS Comput Biol. 2015 Jun 12;11(6):e1004331. doi: 10.1371/journal.pcbi.1004331 (PMC4466596; doi:10.1371/journal.pcbi.1004331)

**S4 Fig. Decision distributions and mean reaction times of the whole sample and corresponding model fits.** To demonstrate that also the group-averaged behavior (often considered in cognitive and neurocognitive research) is well captured by the current model, we here report also averages of empirical as well as fitted performance data. Behavioral data of n=20 subjects are shown in black, simulated data generated from fitted models is shown in orange. Error bars represent the standard error of the mean.

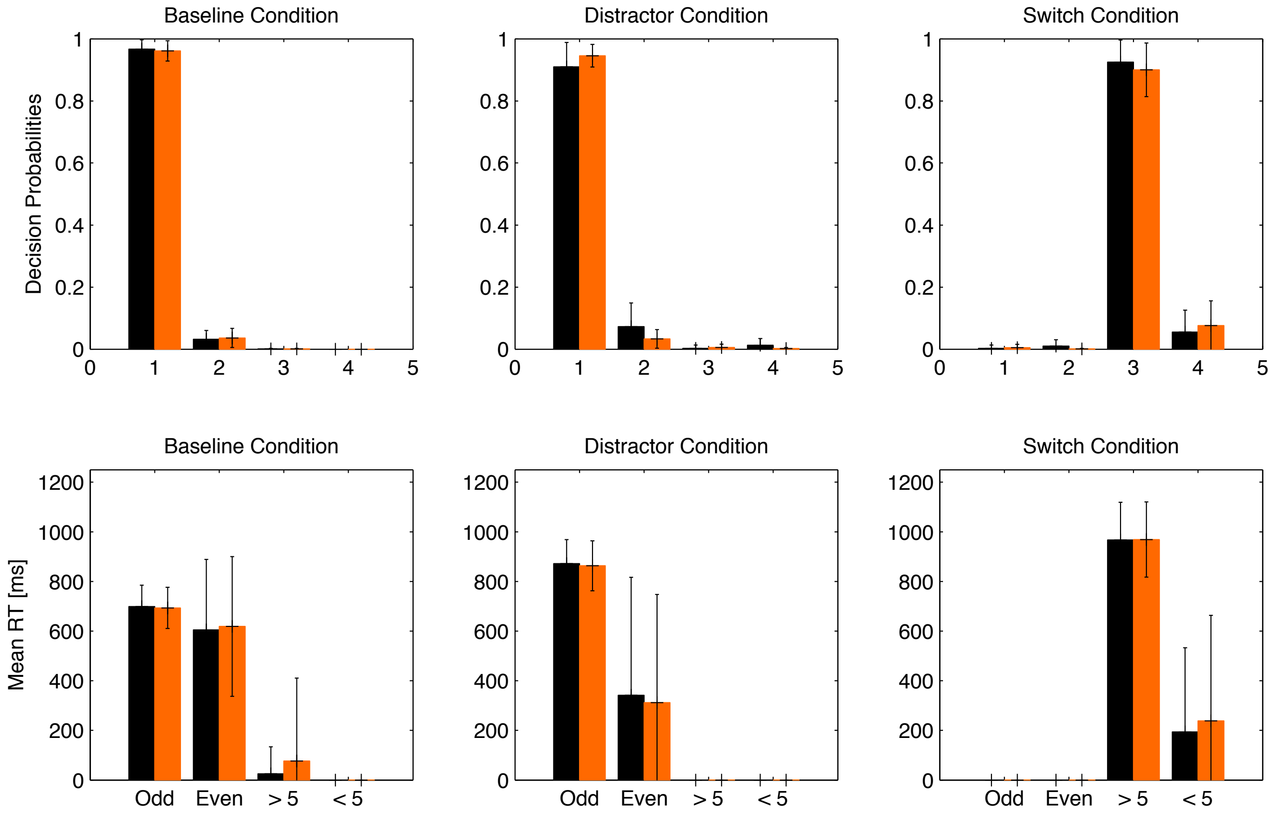

Supplement: S4 Fig — To demonstrate that also the group-averaged behavior (often considered in cognitive and neurocognitive research) is well captured by the current model, we here report also averages of empirical as well as fitted performance data. Behavioral data of n = 20 subjects are shown in black, simulated data generated from fitted models is shown in orange. Error bars represent the standard error of the mean. (PDF) [file pcbi.1004331.s004.pdf]
